# Supplementary material for: Gradient matters via filament diameter-adjustable 3D printing
Source: Nat Commun. 2024 Apr 4;15:2930. doi: 10.1038/s41467-024-47360-y (PMC10994943; doi:10.1038/s41467-024-47360-y)
Supplement: Supplementary file 1 — Supplementary Information [file 41467_2024_47360_MOESM1_ESM.pdf]

Supplementary Information for

Gradient matters via filament diameter-adjustable 3D printing

Huawei Qu[1,2], Chongjian Gao[1], Kaizheng Liu[1], Hongya Fu[2], Zhiyuan Liu[3,4], Paul H. J. Kouwer[5], Zhenyu Han[2] & Changshun Ruan[1,4*]*

[1] Research Center for Human Tissue and Organ Degeneration, Institute of Biomedicine and Biotechnology, Shenzhen Institute of Advanced Technology, Chinese Academy of Sciences, Shenzhen 518055, China.

[2] School of Mechatronics Engineering, Harbin Institute of Technology, Harbin 15000, China.

[3] Research Center for Neural Engineering, Shenzhen Key Laboratory of Smart Sensing and Intelligent Systems, Shenzhen Institute of Advanced Technology, Chinese Academy of Sciences, Shenzhen 518055, China.

[4] University of Chinese Academy of Sciences, Beijing 100049, China.

[5] Institute for Molecules and Materials, Radboud University, Nijmegen 6525 AJ, The Netherlands.

* e-mail: hanzy@hit.edu.cn (Z.H.); cs.ruan@siat.ac.cn (C.R.)

Supplementary information

Supplementary Notes 1 to 4

Supplementary Figures 1 to 23

Supplementary Tables 1 to 6

Supplementary References 1-17

Supplementary Notes

Supplementary Note 1

“CFD simulation result” vs. “weighing result” vs. “imaging result”

In this study, we developed a CFD model using COMSOL Multiphysics 6.0 to simulate the fluid flow of viscoelastic ink inside the nozzle and tip. After determining processing parameters and ink rheology, we substituted the average feeding velocity (v) obtained from the CFD simulation model into Equation (2) (i.e., $Q = v\times\pi d^2/4$), resulting in the flow rate ($Q_{\text{simulation}}$), which we defined as the CFD simulation result, as depicted in Fig. 2a,b. In addition, we plotted the printing velocity V as a function of the filament diameter D using Equation (1) (i.e., $D = (4Q_{\text{simulation}}/V\pi)^{0.5}$), with $Q_{\text{simulation}}$ = 2.963 mm^3/s, as shown in Fig. 2b. Details of the CFD simulation are provided in the “*CFD simulation of ink flow*” of the Methods section.

For weighing results in Fig. 2b, the flow rate Q_{weighing} was measured by weighing ink extruded from the nozzle over a certain time ($Q = m/(\rho\times t)$), where m is the weight of the extrusion ink in t time, ρ is the ink density, and t is the extrusion time. For polymer inks L-PCL and H-PCL, the extrusion times are $t_{\text{L-PCL}}$ = 3 minutes and $t_{\text{H-PCL}}$ = 8 minutes, respectively. The densities of the inks L-PCL and H-PCL were $\rho_{\text{L-PCL}}$ = 1.304 g cm^{-3} and $\rho_{\text{H-PCL}}$ = 1.217 g cm^{-3}, respectively. The flow rate Q_{weighing} obtained by this method is referred to as the weighing result, as shown in Fig. 2a,b. In addition, we plotted the printing velocity V as a function of the filament diameter D using Equation (1) (i.e., $D = (4Q_{\text{weighing}}/V\pi)^{0.5}$), where Q_{weighing} = 1.240 mm^3/s, as shown in Fig. 2b. Further details can be found in the “*Measurement of flow rate*” of the Methods section.

For imaging results in Fig. 2b, constant-diameter D filament samples were 3D printed at different printing velocities V (0.5, 1, 2, 3, 4, 5, 6, 7, 8, 9, 10, 11, 12, 13, and 14 mm s^{-1}), followed by cutting with a knife. Subsequently, filament cross sections were photographed using a light microscope (HiROX MXB-5040RZ, Japan). The cross-sectional area S was determined through ImageJ software (Fiji Is Just ImageJ, https://imagej.net/software/fiji/). Filament diameters D corresponding to the various printing velocities V were computed using the formula $D = (4S/\pi)^{0.5}$. These results (data points) are denoted as imaging results in Fig. 2b. Further details are provided in the “*Filament cross-section quantification*” of the Methods section.

Supplementary Note 2

Printing height *H* vs. ideal filament diameter *D* for keeping general deposition state

In the map of filament deposition states, the acceptable general state was marked as solid triangles (Fig. 2c and Supplementary Figs. 8-9). To establish the relationship between the printing height H and the filament diameter D, we performed the following step-by-step derivation.

(1) The transformation of Equation (2) (i.e., $Q = v \times \pi d^2/4$) is performed to obtain the following equation:

$$v = 4Q/\pi d^2 \qquad \text{(S1)}$$

where Q is the extrusion flow rate (mm^3/s), v is the average feeding velocity through the nozzle tip (mm/s), d is the inner diameter of the nozzle tip's outlet (mm), and π (Pi) is the ratio of a circle's circumference to its diameter.

(2) $H^* = H/d$ and $V^* = V/v$ are substituted into Equation (3) (i.e., $H^* = \text{k} \times (V^*)^{-0.5}$) to remove the dimensionless parameters H^* and V^*. The new equation is shown below.

$$H/d = \text{k} \times (V/v)^{-0.5} \qquad \text{(S2)}$$

(3) Equation (S1) (i.e., $v = 4Q/\pi d^2$) is substituted into Equation (S2) (i.e., $H/d = \text{k} \times (V/v)^{-0.5}$) to remove the average ink feeding velocity (i.e., v) through the nozzle tip. The new equation is shown below.

$$H/d = \text{k} \times [V/(4Q/\pi d^2)]^{-0.5} \qquad \text{(S3)}$$

Equation (S3) above is adjusted and simplified to obtain the following equation.

$$H = \text{k} \times (4Q/V\pi)^{0.5} \qquad \text{(S4)}$$

(4) Equation (1) (i.e., $D = (4Q/V\pi)^{0.5}$) is switched left and right (i.e., $(4Q/V\pi)^{0.5} = D$), and then substituted into Equation (S4) above (i.e., $H = \text{k} \times (4Q/V\pi)^{0.5}$) to remove the constant, Q, V and π. The new equation about H and D is shown below.

$$H = \text{k} \times D \qquad \text{(S5)}$$

Equation (S5) above is also shown in the Results section of the main text as Equation (4).

Supplementary Note 3

"Fabrication resolution" vs. "fabrication G-codes" vs. "its file size"

The commercially available software Rhinoceros and its tool Grasshopper were used to plot and plan the printing trajectory in the FDA-3DP strategy. Using this software, the printing trajectory (2D lines in the *x*-*z* or y-*z* plane) was equally segmented according to the fabrication resolution of 1.25 mm using the Grasshopper command "divide". The obtained equal points with *x*-*y*-*z* coordinates (*z* coordinate value is *H*) and their corresponding *V* were written as G-codes after adding the code start (initialization) and end commands (Supplementary Fig. 12). Notably, the fabrication resolution determines the number of aliquots, and then affects the file size of the G-codes, as shown in Fig. 2e-f. In this work, the fabrication resolutions of 10, 5, 2.5, 1.25, and 0.625 mm correspond to the divided numbers of 5, 9, 17, 33, and 65, respectively (Supplementary Fig. 11).

Supplementary Note 4

"Filament cross-section" vs. "printing velocity *V*"

The viscoelastic ink deposited by 3D printing is fluid and the filament cross-section is nearly oblong, as shown in Supplementary Fig. 3a-m. The relationship of *V* and *AR* are fitted ($AR = 2.457\times V^{-0.297}$), as shown in Supplementary Fig. 3n. According to the definition of *AR*, we get $AR = l_w/l_h$ and $S = l_w/l_h\text{-}l_h^2\,(1\text{-}\pi/4)$ (Supplementary Fig. 3o). Based on Equation (1), we get $S = Q\times V^{-1}$. After determining the processing parameters (nozzle tip's inner diameter of 400 μm from Hangzhou Regenovo Biotechnology Co., Ltd., extrusion air pressure of 500 kPa) and ink rheology (H-PCL), the ink flow rate $Q_{simulation} = 2.963\ mm^3/s$ is obtained from the CFD simulation model (Fig. 2b). In addition, *V* for a series of movement points on the printing trajectory can be determined based on the gradient in pore sizes. Finally, the width l_w and height l_h at different *V* are obtained.

Supplementary Figures

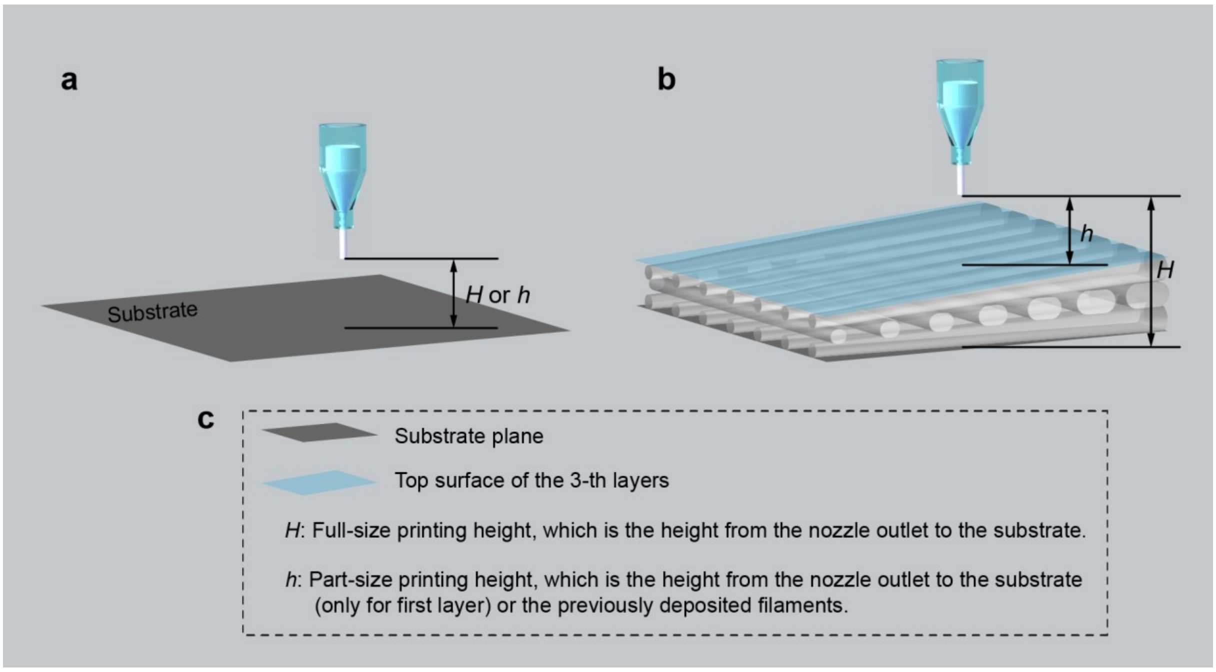

Supplementary Fig. 1 | Definition of *H* and *h*. **a**, Schematic diagram of *H* and *h* in the only first layer condition. **b**, Schematic diagram of *H* and *h* in the multiple layer condition. **c**, Annotations for Supplementary Fig. 1a and b. *H* is the height from the nozzle outlet to the substrate. *h* is the height from the nozzle outlet to the substrate (only for the first layer) or the previously deposited filaments.

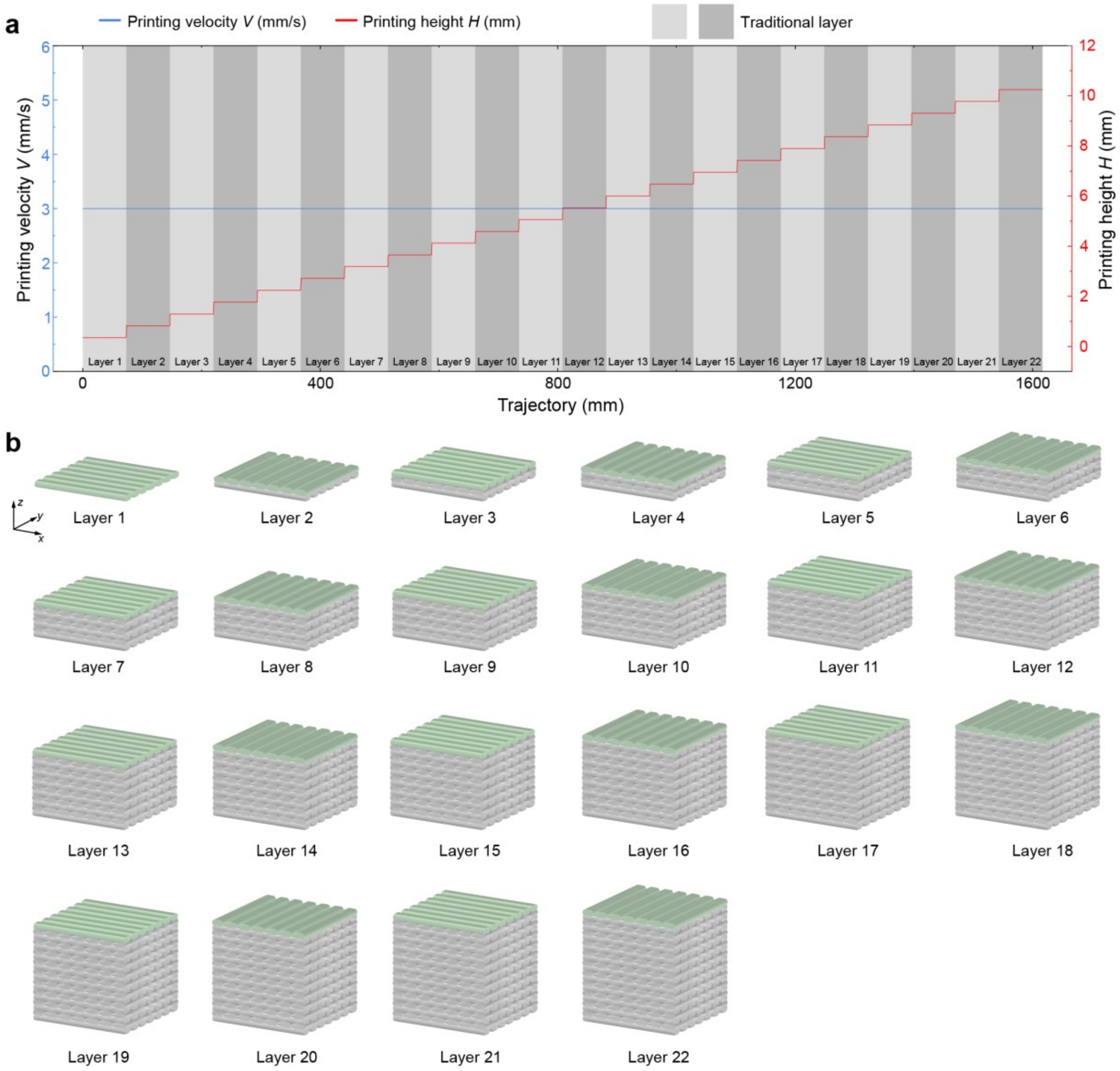

Supplementary Fig. 2 | Phase diagram of printing parameters and filament stacking models of homogeneous porous sample created via traditional-3DP strategy. **a**, Details of *V* and *H*. *V* and *H* are fixed during the intra-layer printing process. *H* is the distance from the nozzle outlet to the substrate. **b**, A series of uniform pore models with constant *D* and cross-sectional shape (width l_w and height l_h). The final model was constructed by stacking 22-layer filaments.

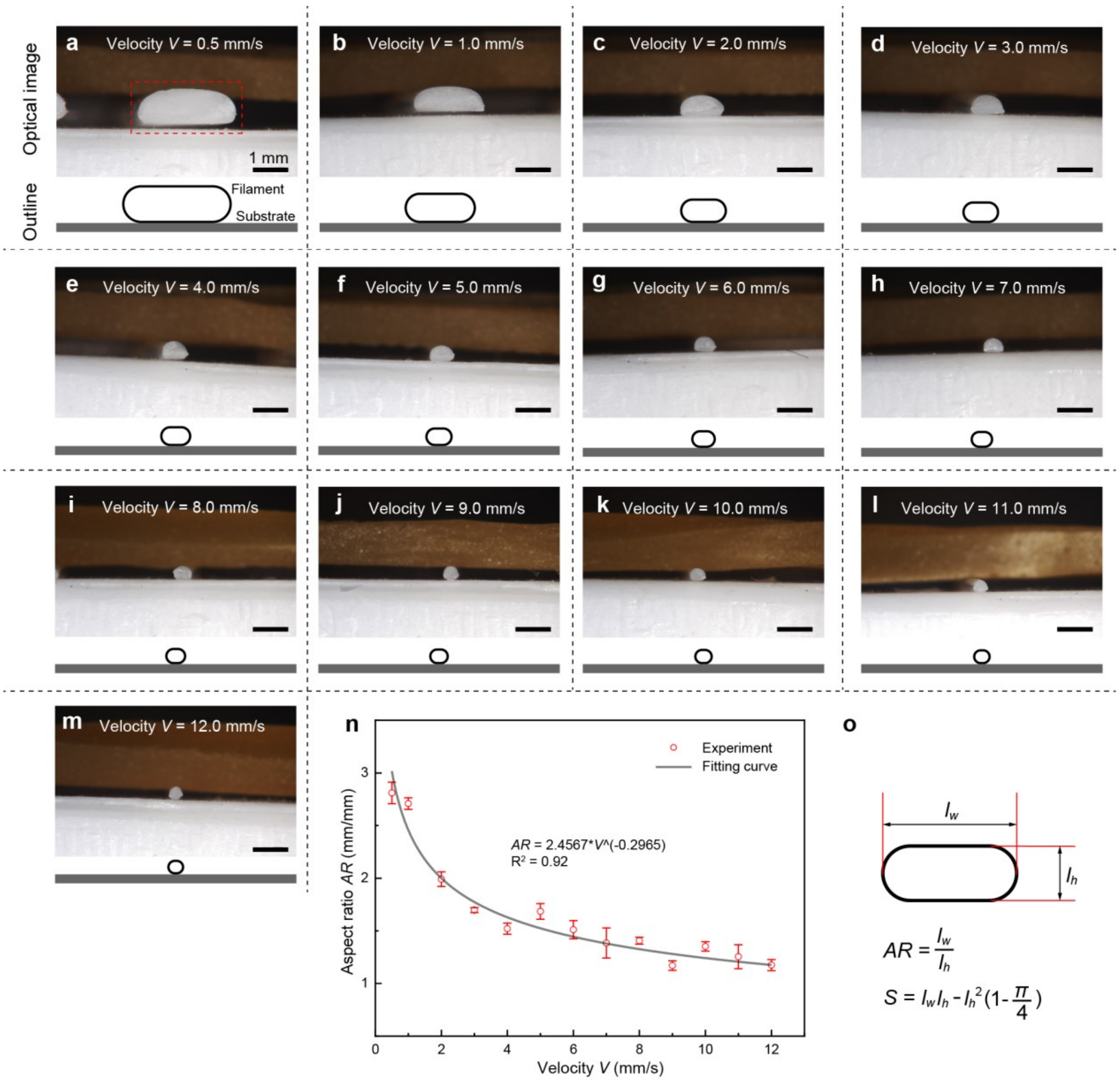

Supplementary Fig. 3 | H-PCL filament cross-sections at different printing velocities *V* at *Q* = 3.748 mm^3/s (a-m), the relationship between printing velocity *V* and aspect ratio *AR* (n), and schematic diagram of oblong filament cross section (o). *AR* gradually decreases as the printing velocity *V* increases. Data is mean ± standard error (means ± s.d., n = 4). The filament cross-section is nearly oblong, and its key parameters are cross-sectional width l_w and height l_h. The following functions can be obtained: aspect ratio $AR = l_w/l_h$ and cross-sectional area $S = l_w \times l_h - l_h^2(1-\pi/4)$.

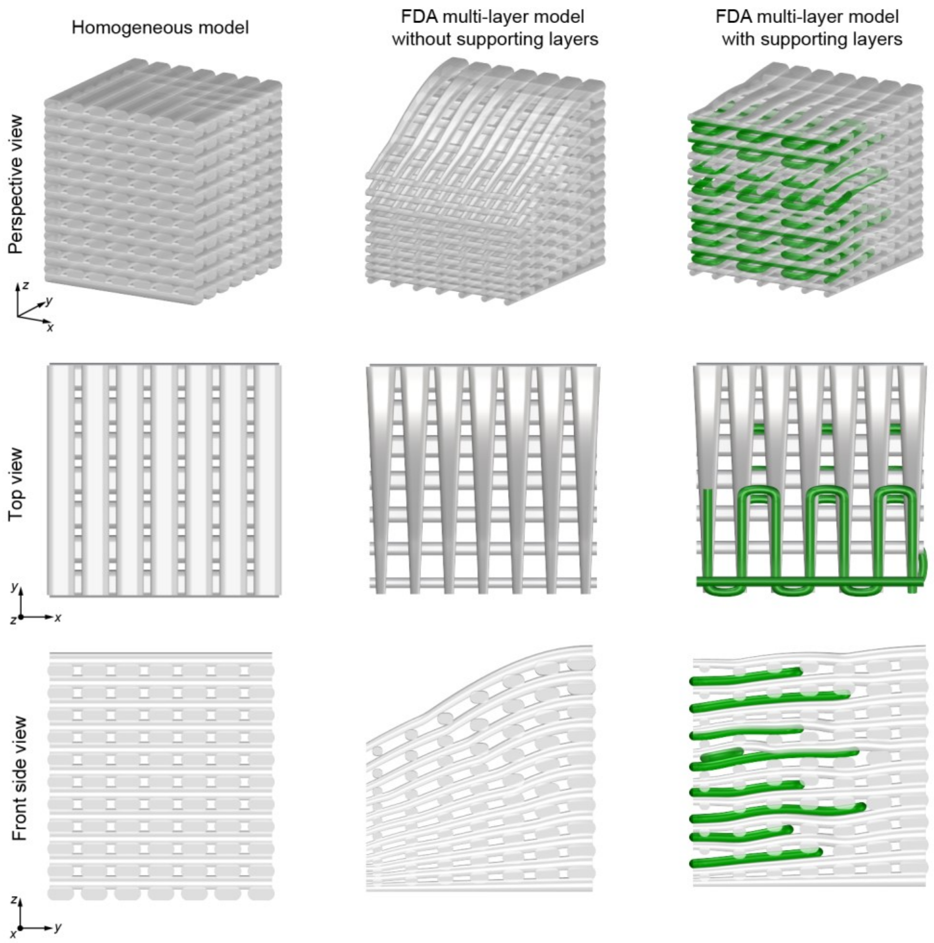

Supplementary Fig. 4 | Details of the homogeneous model (left) and horizontal gradient pore models without (middle) and with (right) supporting layers. The traditional-3DP strategy is limited by the fixed D and the pore size is the same everywhere (left). In our proposed strategy, if it is not supported (middle), the horizontal gradient model has a collapse in the large pore region. Therefore, we added supporting layers with a constant minimum D_{min} (right, green filament model).

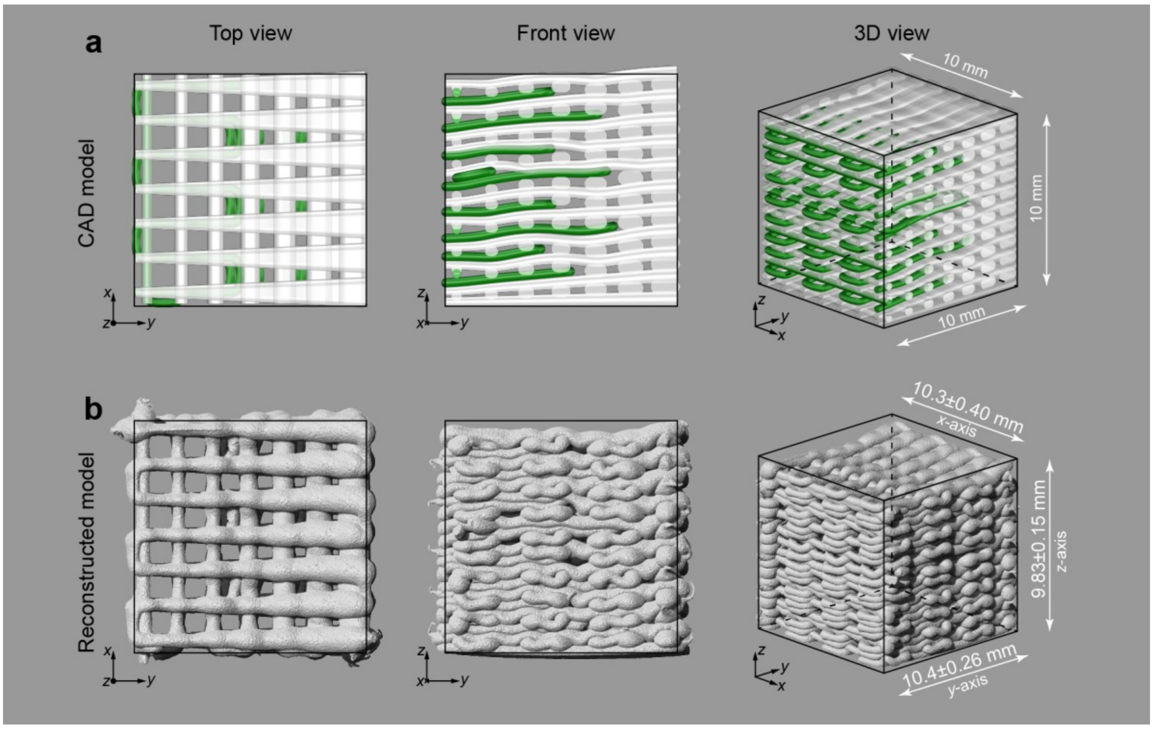

Supplementary Fig. 5 | Dimensional results of the horizontal gradient CAD model and corresponding printed samples. **a**, Cube boundary (10 mm×10 mm×10 mm) of the CAD model presented in Fig. 1d(i-ii). **b**, Dimensional measurements of the printed samples depicted in Fig. 1d(iii) along the *x*, *y*, and *z* directions are 10.3±0.40 mm, 10.4±0.26 mm, and 9.83±0.15 mm, respectively.

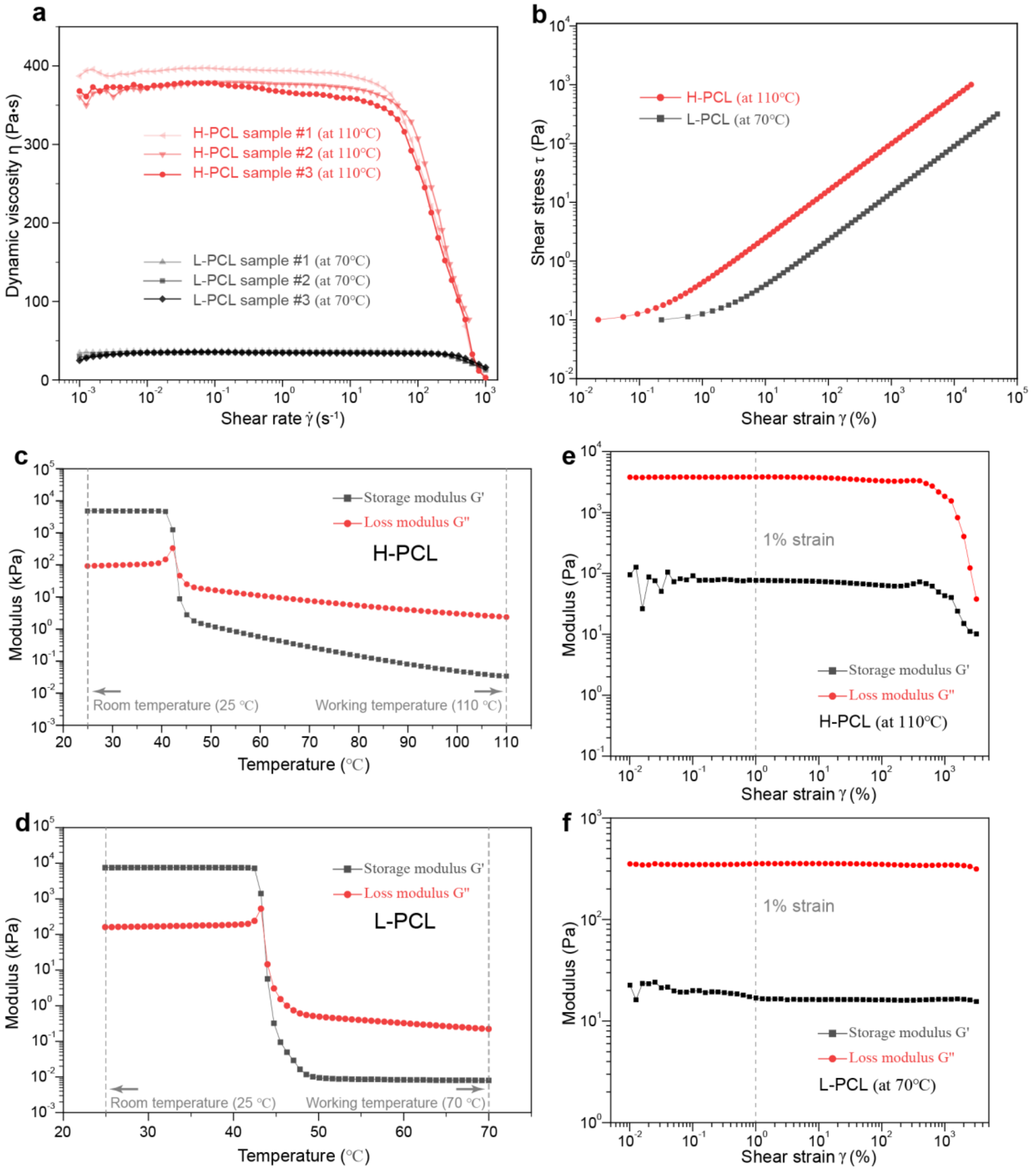

Supplementary Fig. 6 | Rheological characteristics of the polymer inks (L-PCL and H-PCL) used in this study. **a**, Dynamic viscosity of L-PCL and H-PCL at 70 °C and 110 °C, respectively. Both L-PCL and H-PCL exhibit non-Newtonian behavior, demonstrating the shear-thinning phenomenon at a high shear rate (about 10^2 s^{-1}). Three repetitions were conducted. The shear rate ranges from 10^{-3} s^{-1} to 10^3 s^{-1}. The zero-shear viscosities η_0 of L-PCL and H-PCL are approximately 382.299 Pa·s and 36.323 Pa·s, respectively. **b**, Relationship between shear stress and shear strain using rotational tests with controlled shear stress (CSS). Both L-PCL and H-PCL exhibit the yield stress. **c**, Storage *G*' and loss *G*'' modulus of the H-PCL ink for temperature scanning (from 110 °C to 25 °C) through oscillatory measurement. **d**, Storage *G*' and loss *G*''

modulus of the L-PCL ink for temperature scanning (from 70 ℃ to 25 ℃) through oscillatory measurement. During material deformation, elasticity, and viscosity are measured by the storage modulus *G*' and loss modulus *G*'', respectively. **e**, Storage *G*' and loss *G*'' modulus versus strain γ of the H-PCL ink for the amplitude sweep (from 0.01% to 3000%) through oscillatory measurement at 110 °C working temperature. **f**, Storage *G*' and loss *G*'' modulus versus strain (γ) of the L-PCL ink for the amplitude sweep (from 0.01% to 3000%) through oscillatory measurement at 70 °C working temperature.

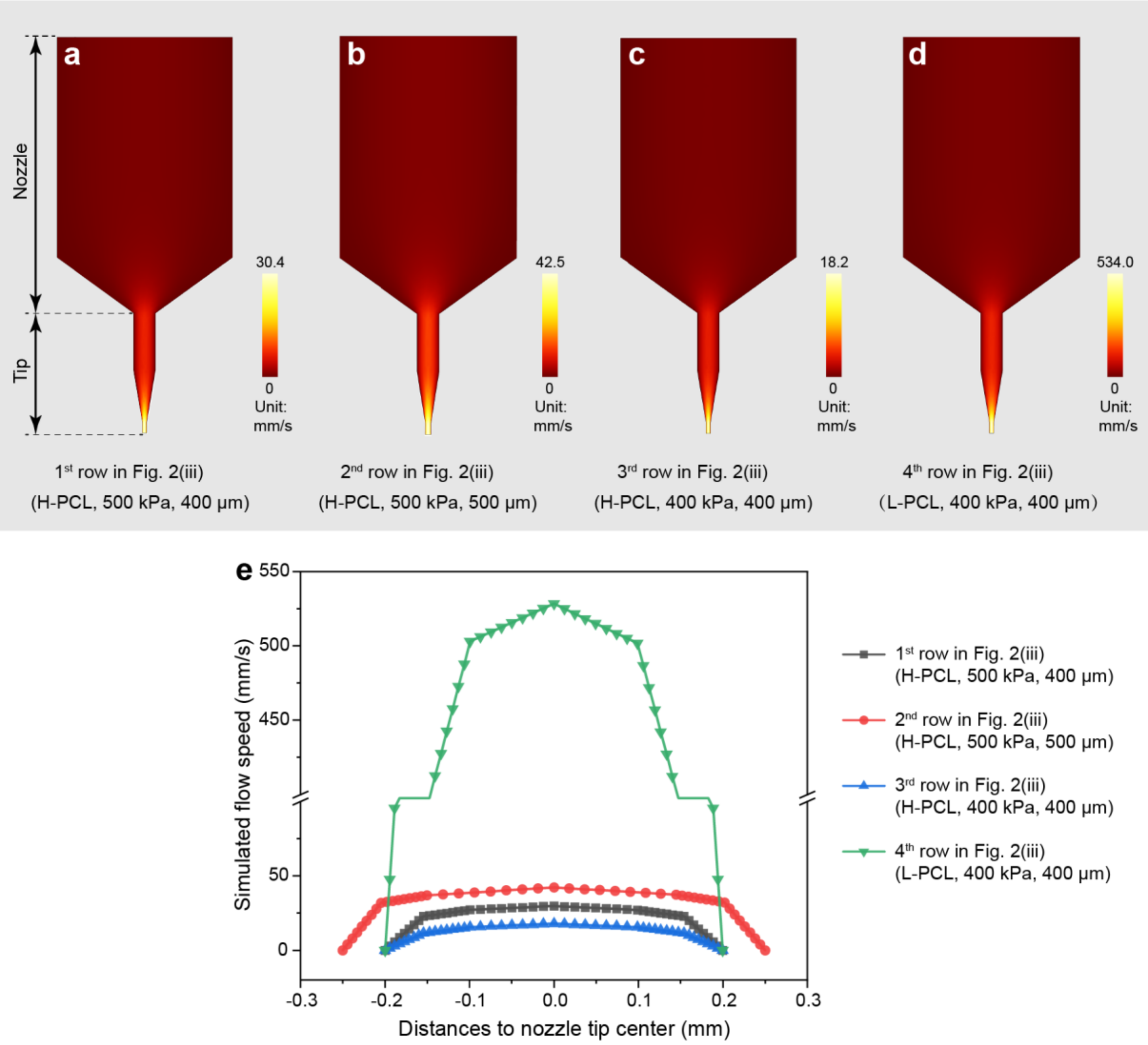

Supplementary Fig. 7 | Flow velocity distribution in CFD simulation. **a**, Flow velocity distribution in 1st row of Fig. 2(iii). Processing parameters include H-PCL ink, extrusion air pressure of 500 kPa, and nozzle tip inner diameter of 400 μm. **b**, Flow velocity distribution in 2nd row of Fig. 2(iii). Processing parameters include H-PCL ink, extrusion air pressure of 500 kPa, and nozzle tip inner diameter of 500 μm. **c**, Flow velocity distribution in 3rd row of Fig. 2(iii). Processing parameters include H-PCL ink, extrusion air pressure of 400 kPa, and nozzle tip inner diameter of 400 μm. **d**, Flow velocity distribution in 4th row of Fig. 2(iii). Processing parameters include L-PCL ink, extrusion air pressure of 500 kPa, and nozzle tip inner diameter of 400 μm. **e**, Quantification of flow velocity at the nozzle tip's outlet.

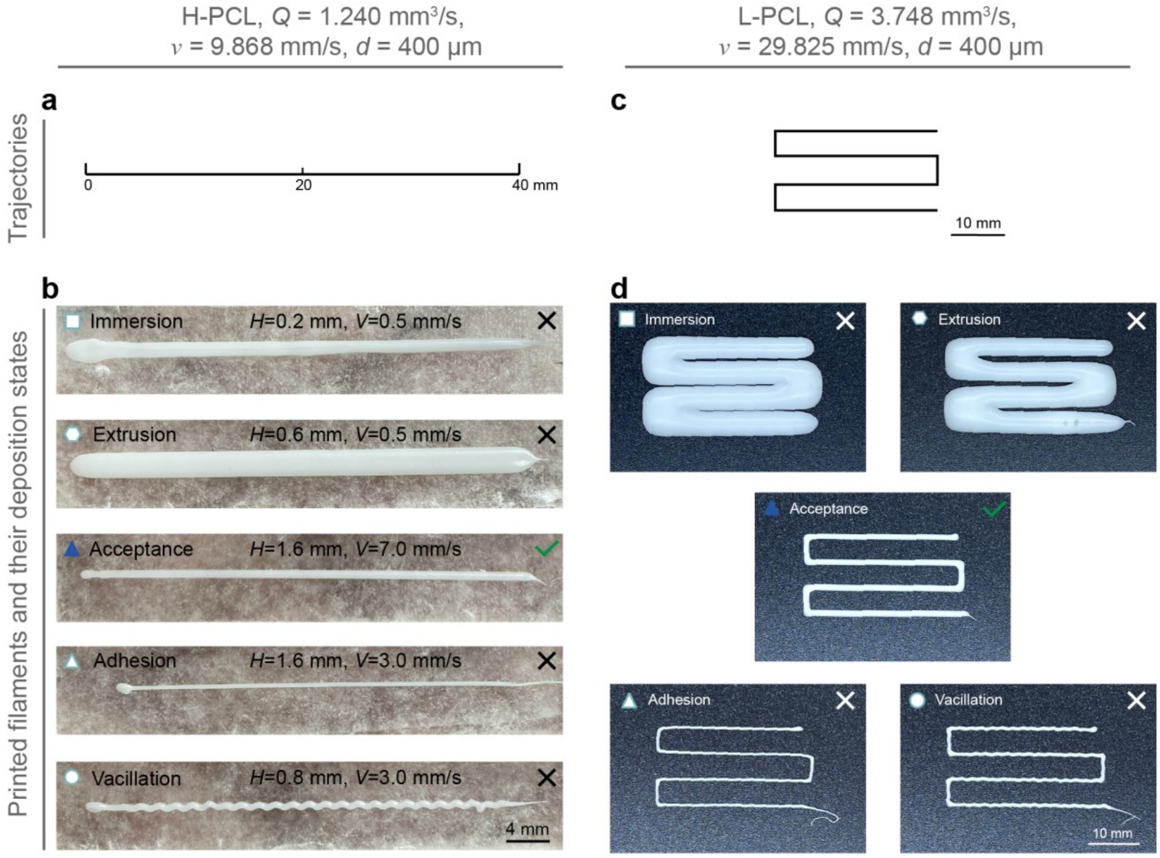

Supplementary Fig. 8 | Printing trajectories and top views of printed H-PCL and L-PCL filaments with five deposition states. **a** and **c**, Printing trajectories of Condition 1 (a) and Condition 2 (c). **b** and **d**, 3D printed single-filament samples with different V and H for Condition 1 (b) and Condition 2 (d), showing five deposition states (immersion, extrusion, acceptance, adhesion, and vacillation). In Condition 1 (a and b), the print ink is H-PCL, the flow rate is Q_{weighing} = 1.240 mm^3/s, the average feeding velocity is v= 9.868 mm/s, and the inner diameter of the nozzle tip #1 (from Hangzhou Regenovo Biotechnology Co., Ltd., China) is 400 μm. In Condition 2 (c and d), the print ink is L-PCL, the flow rate is Q = 3.748 mm^3/s, the average feeding velocity is v = 29.825 mm/s, and the inner diameter of the nozzle tip #2 (from Qingyuan Fuying Electronics Co., Ltd., China) is 400 μm.

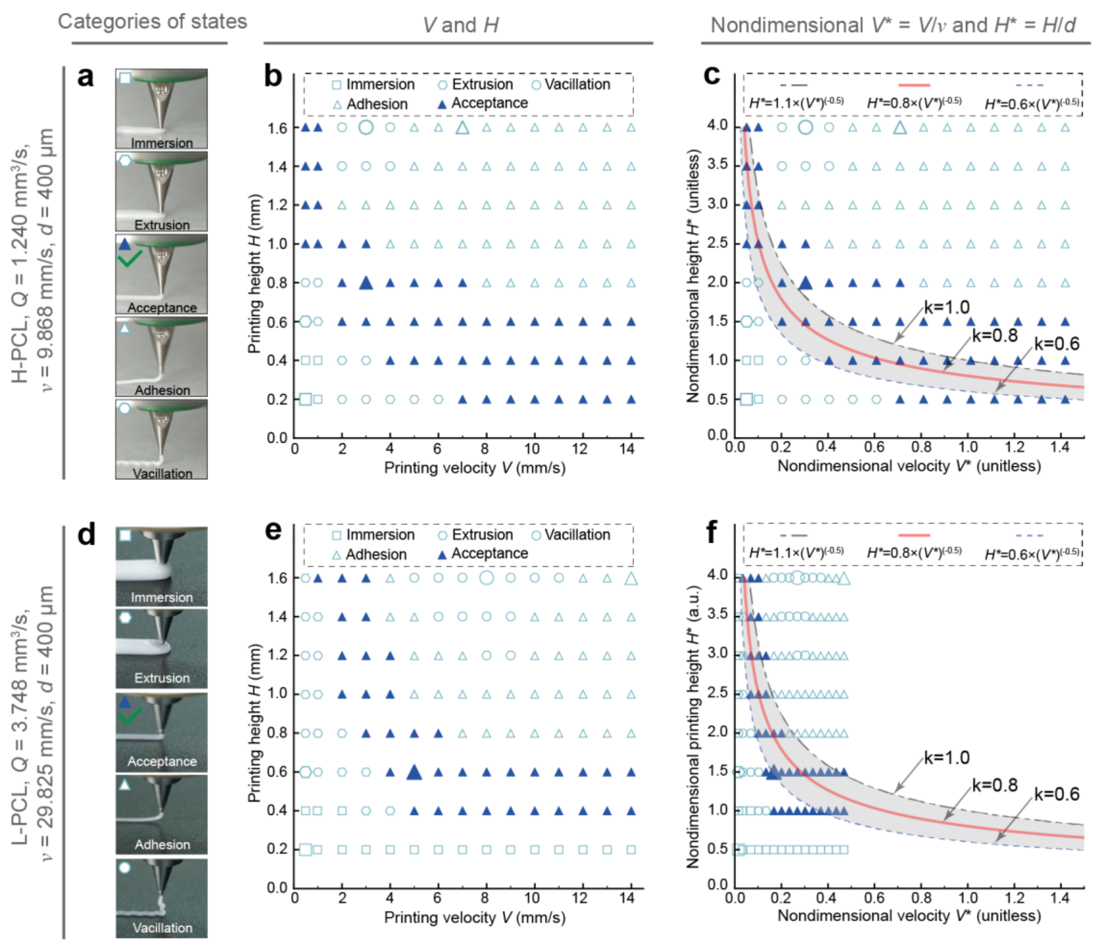

Supplementary Fig. 9 | Effect of printing parameters *V* and *H* on filament deposition state at different flow rates *Q*. **a** and **d**, Categories of filament deposition states. **b** and **e**, Key printing parameters *V* and *H* on deposition state. **c** and **f**, Nondimensional *V** and *H** on deposition state, where *V** = *V*/*v* and *H** = *H*/*d*. **a-c**, the working condition is H-PCL ink, extrusion flow rate of Q_{weighing} = 1.240 mm^3/s, ink feeding velocity of *v*= 9.868 mm/s, and nozzle tip #1 (from Hangzhou Regenovo Biotechnology Co., Ltd., China) inner diameter of 400 μm. **d-f**, the working condition is L-PCL ink, extrusion air pressure of 400 kPa, extrusion flow rate of Q_{weighing} = 3.748 mm^3/s, ink feeding velocity of *v*= 29.825 mm/s, and nozzle tip #2 (from Qingyuan Fuying Electronics Co., Ltd., China) inner diameter of 400 μm. The filament deposition states include immersion, extrusion, acceptance, adhesion, and vacillation. The fitting of *H** and *V** corresponding to the acceptable deposition state results in Equation (3) (i.e., $H^* = \text{k}\times(V^*)^{-0.5}$), where k ranges about from 0.6 to 1.0 (gray banded area in sub-figure **c** and **f**). We chose k = 0.8 in this study.

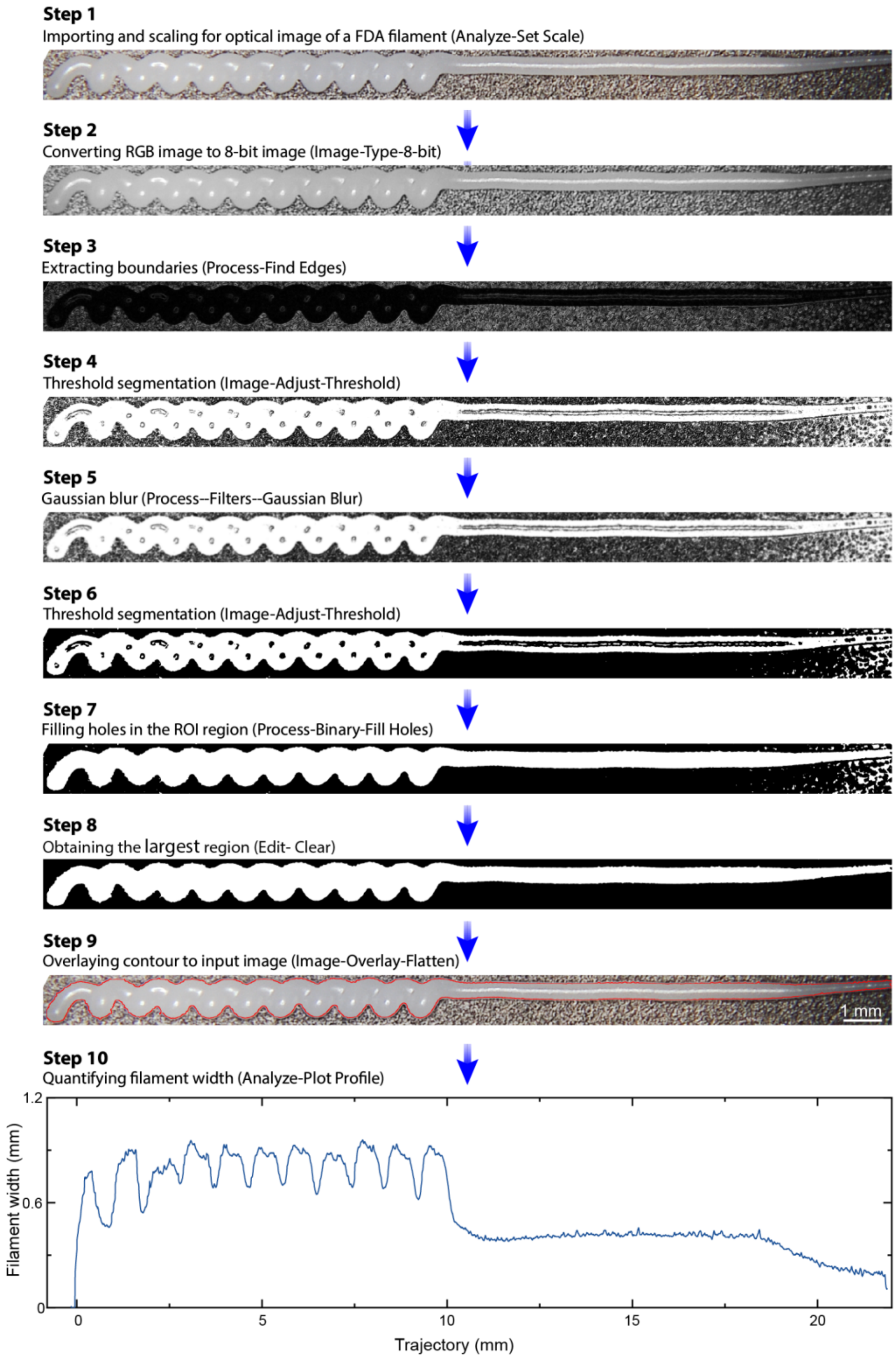

Supplementary Fig. 10 | Steps for quantifying filament width l_w using software ImageJ.

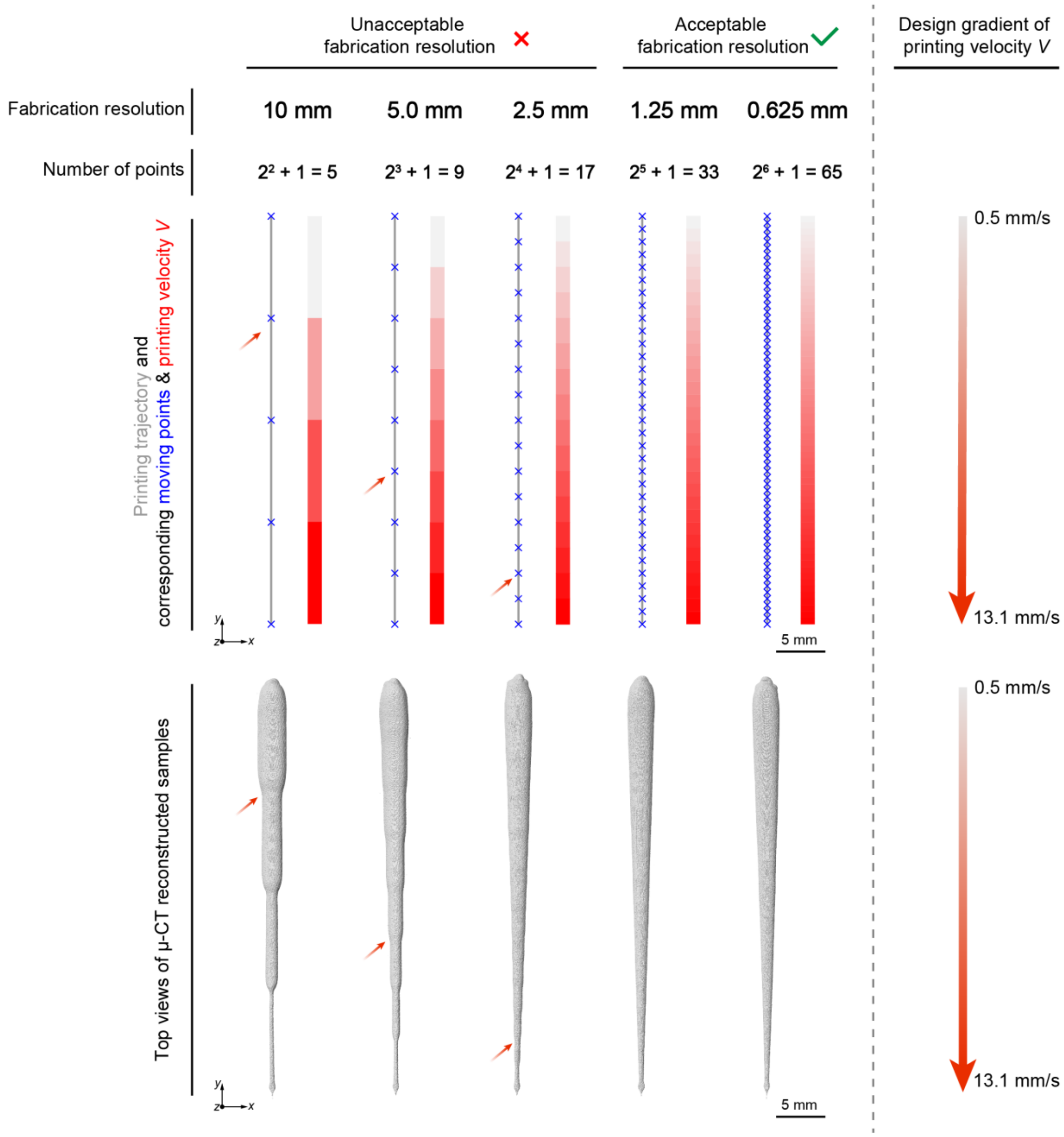

Supplementary Fig. 11 | Printing trajectory, number of moving points, printing velocity, and top views of the μ-CT reconstructed 40 mm-long FDA samples with different fabrication resolution (10, 5.0, 2.5, 1.25, and 0.625 mm). In this figure, the gray line is the 40 mm-long printing trajectory; the blue cross is the movement points; the red rainbow chart is the design gradient of the printing velocity V; and the oblique red arrows show the step phenomenon. As the fabrication resolution increases, the tendency of the filament width l_w to vary uniformly increases significantly, and the step phenomenon (oblique red arrow) gradually disappears.

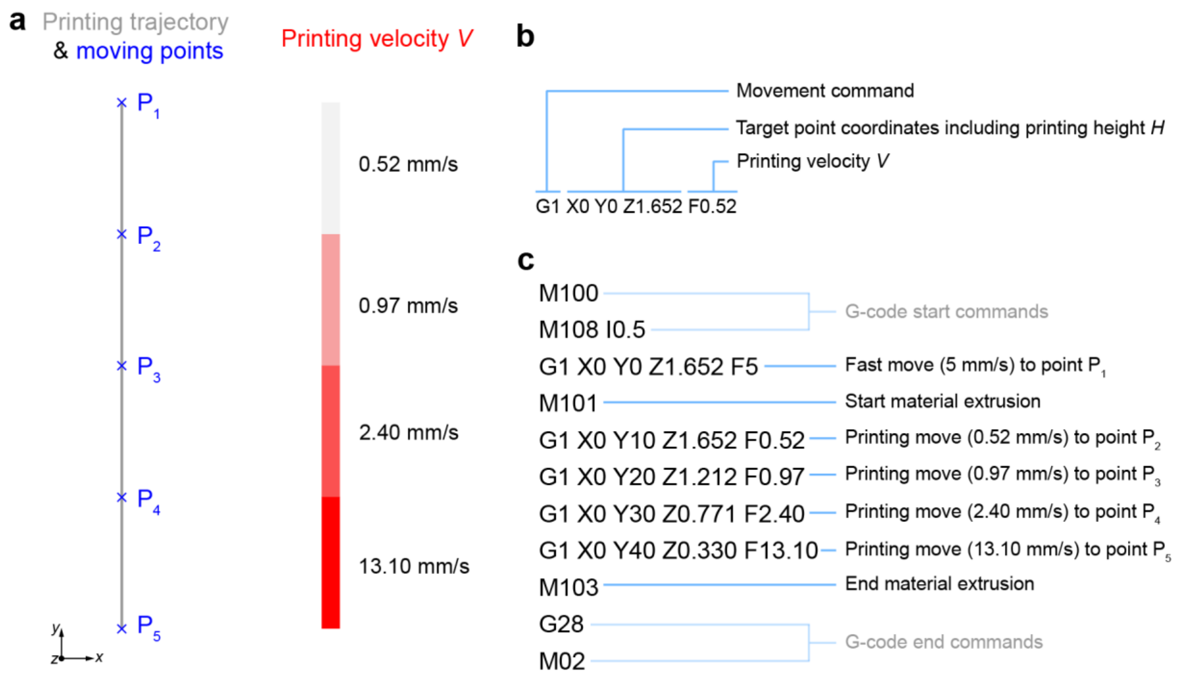

Supplementary Fig. 12 | a, Printing trajectory, moving points, printing velocity of a 40 mm-long FDA filament. **b**, G-code meaning. **c**, Fabrication G-codes for a 40 mm-long FDA filament. The fabrication G-codes consist of a sequence of 3D point coordinates (from P_1 to P_5) and their matching printing velocity *V* (from 0.52 to 13.10 mm/s) and height *H* (from 1.652 to 0.330 mm).

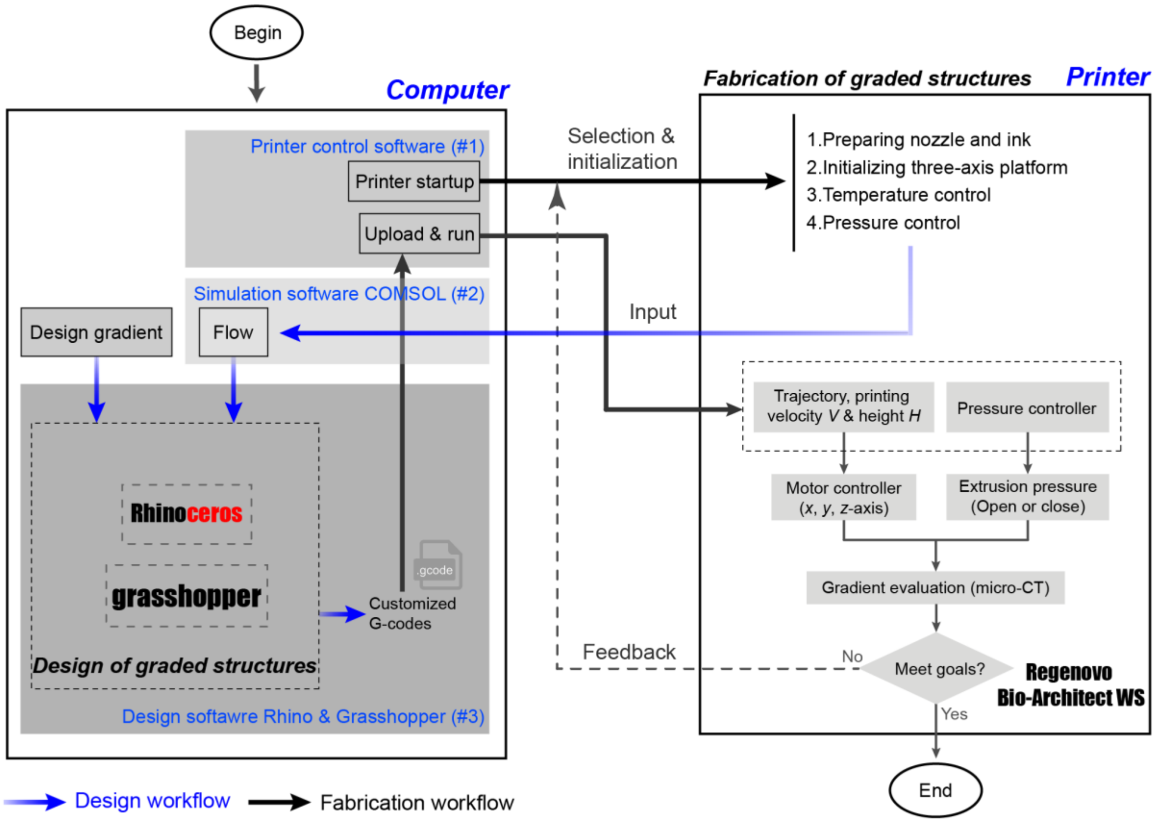

Supplementary Fig. 13 | Design-to-fabrication workflow between the computer and printer used in the FDA-3DP strategy. On the left and right are the operations performed by the computer and printer, respectively. The computer part includes 3D printer's control software (3D Bio-Architect, Regenovo Biotechnology Co., Ltd., China), simulation software (COMSOL Multiphysics 6.0, Dassault Systèmes, USA), and design software (Rhinoceros 3D embedded with a parametric design tool Grasshopper, Robert McNeel & Associates, USA). The extrusion-based 3D printer (Regenovo Bio-Architect WS) is from Regenovo Biotechnology Co., Ltd., China. The blue and black arrows are the forward trajectories of the design and manufacturing parts of this workflow, respectively.

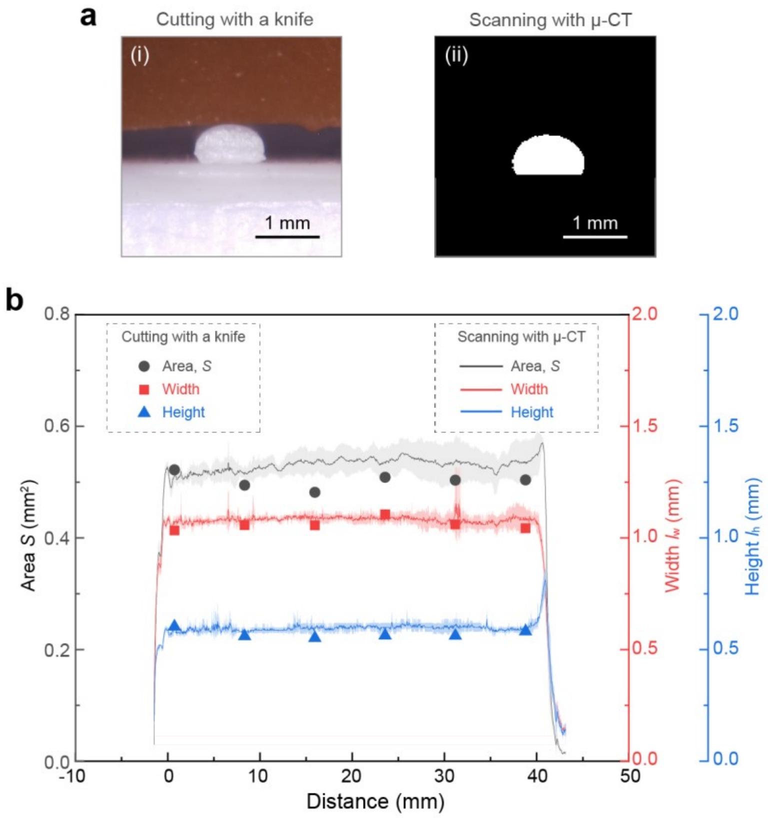

Supplementary Fig. 14 | The agreement of the filament cross sections obtained through knife cutting and μ-CT scanning. **a**, Filament cross section contours were obtained by cutting with a knife and scanning with a μ-CT device (SCANCO, Switzerland). Scale bar, 1 mm. **b**, Quantification of the filament cross sections through cutting and scanning, including area S, width l_w, and height l_h. Three μ-CT scan samples (lines with error bands) and six knife-cut samples (symbolic elements) were prepared and evaluated, respectively.

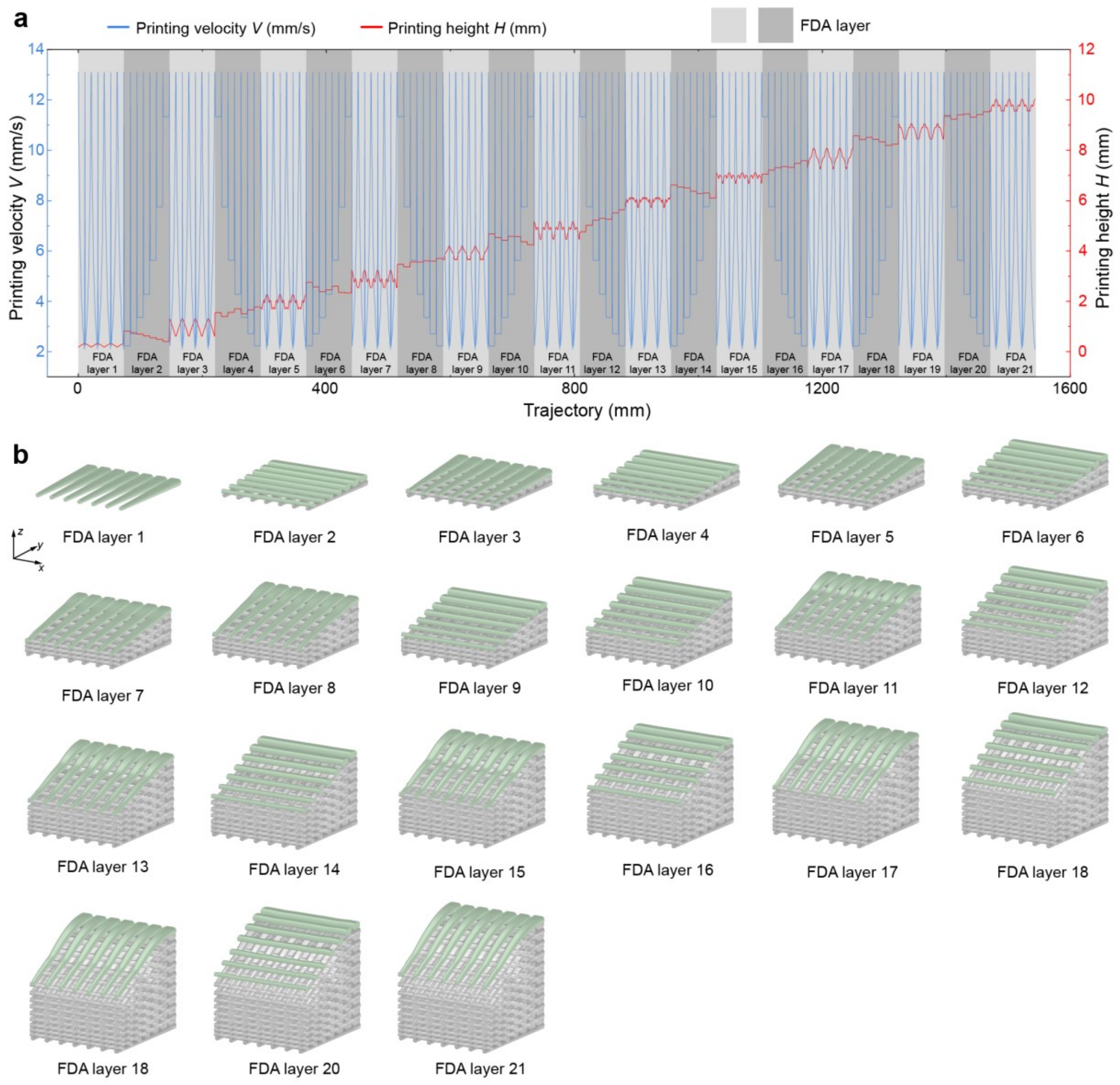

Supplementary Fig. 15 | Phase diagram of printing parameters and filament stacking models of the horizontal gradient model without supporting layers created by FDA-3DP strategy. **a**, Details of *V* and *H*. During printing, *V* and *H* are continuously changed to precisely control the volume of deposited ink on the printing trajectory. **b**, A series of horizontal gradient pore models with variable-*D* FDA layers. The final model was constructed by stacking 21-layer filaments.

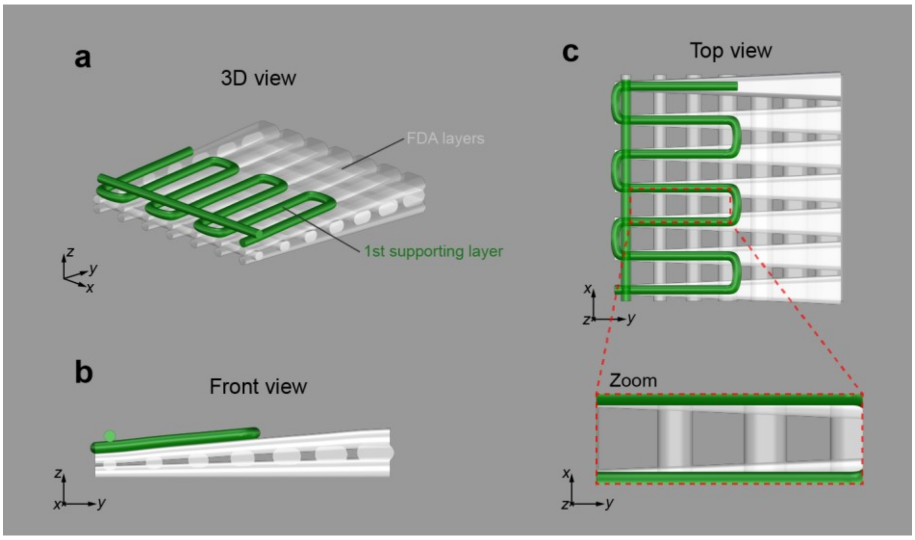

Supplementary Fig. 16 | Supporting layers with a constant diameter ($D_{\min}$) obtained using the simplified compensation mechanism in this study. To simplify the process and reduce workload, we chose the minimum value in the range of filament diameters produced by this work ($D_{\min}$, corresponding to $V_{\max}$) as the constant diameter. In this illustration, the filament model (green) of the 1st supporting layer is stacked onto three FDA layers (light gray) with a horizontal gradient in pore sizes. **a**, 3D view. **b**, Front view. **c**, Top view and its partial enlargement.

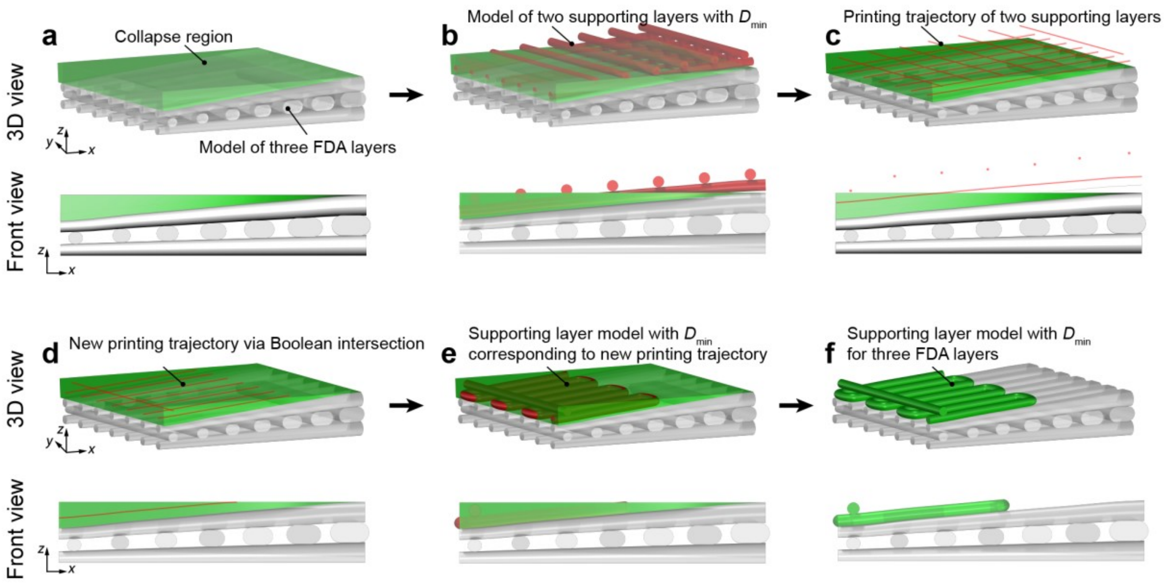

Supplementary Fig. 17 | Thickness compensation mechanism for obtaining the printing trajectory and the 3D model of the supporting layers using Boolean intersection operations. The top and bottom sub-figures are the 3D view and the front view, respectively. **a**, Collapse region (green) for the three FDA layer models (grey). **b**, Model of two supporting layers with $D_{\min}$ (red) are positioned on top of the three FDA layer models (grey). **c**, Printing trajectory corresponding to two supporting layers. **d**, Result of the new printing trajectory obtained by Boolean intersection between the collapsed region and the printing trajectory of two supporting layers. **e**, New supporting layer model with fixed $D_{\min}$ by pipe operation. **f**, Result of supporting layer model for the three FDA layers.

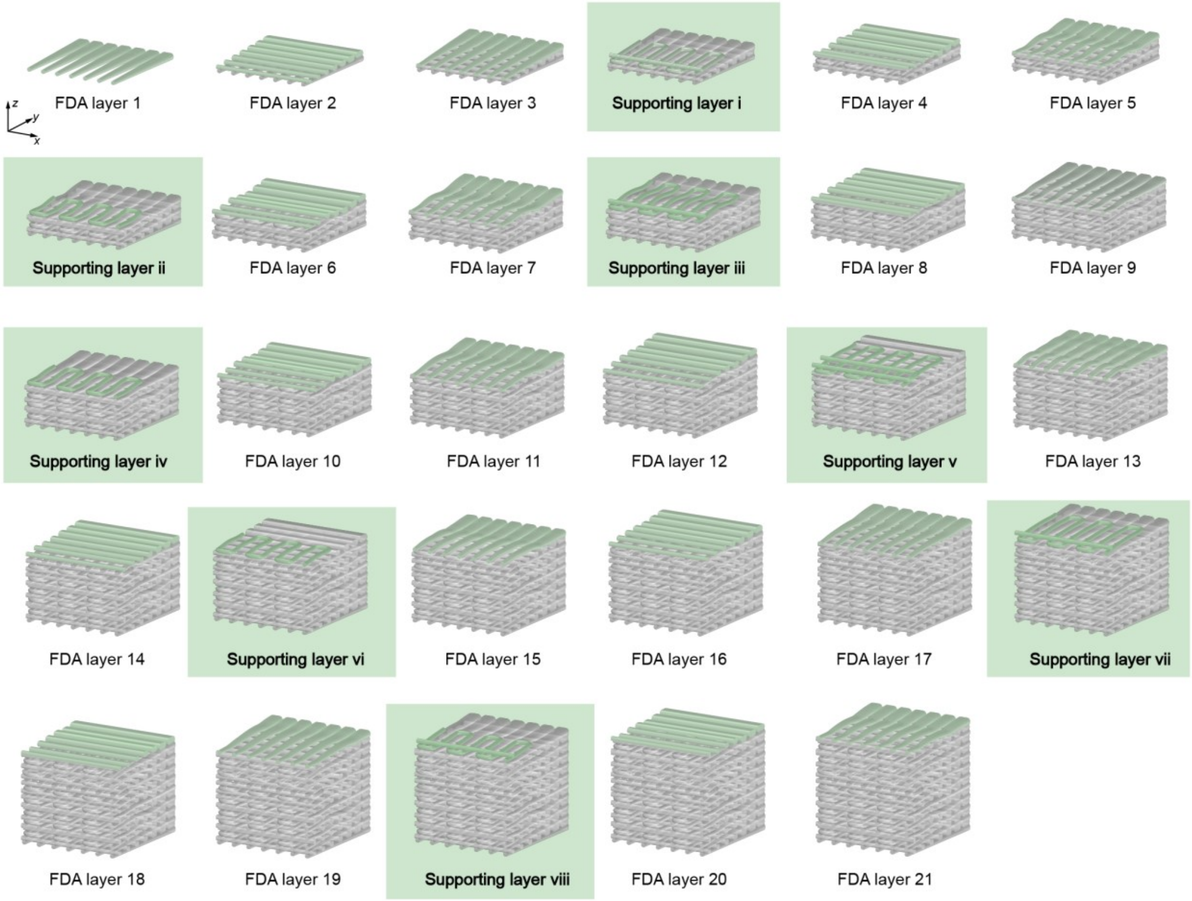

Supplementary Fig. 18 | Filament stacking models of the horizontal gradient pore model with FDA and supporting layers created by FDA-3DP strategy. A series of horizontal gradient pore models with variable-*D* FDA layers and constant-*D* supporting layers. The final model without collapse region was constructed by stacking 21-layer FDA filaments and 8-layer supporting filaments.

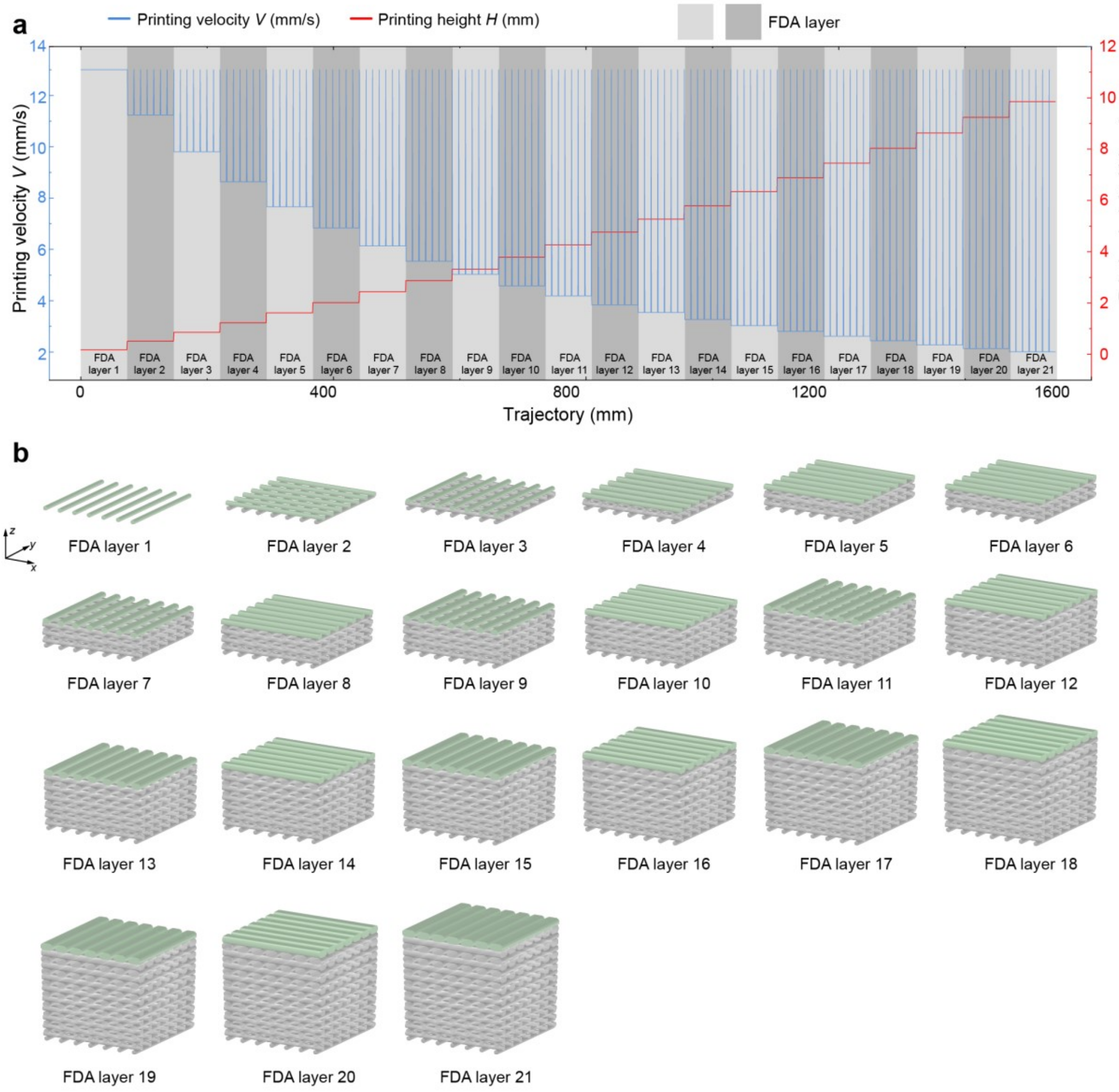

Supplementary Fig. 19 | Phase diagram of printing parameters and filament stacking models of axial gradient model. **a**, Details of *V* and *H*. During the printing process, *V* and *H* gradually increase from bottom to top between layers. However, they remain constant within the layer. **b**, A series of axial gradient pore models with variable-*D* FDA layers. The final model was constructed by stacking 21-layer FDA filaments.

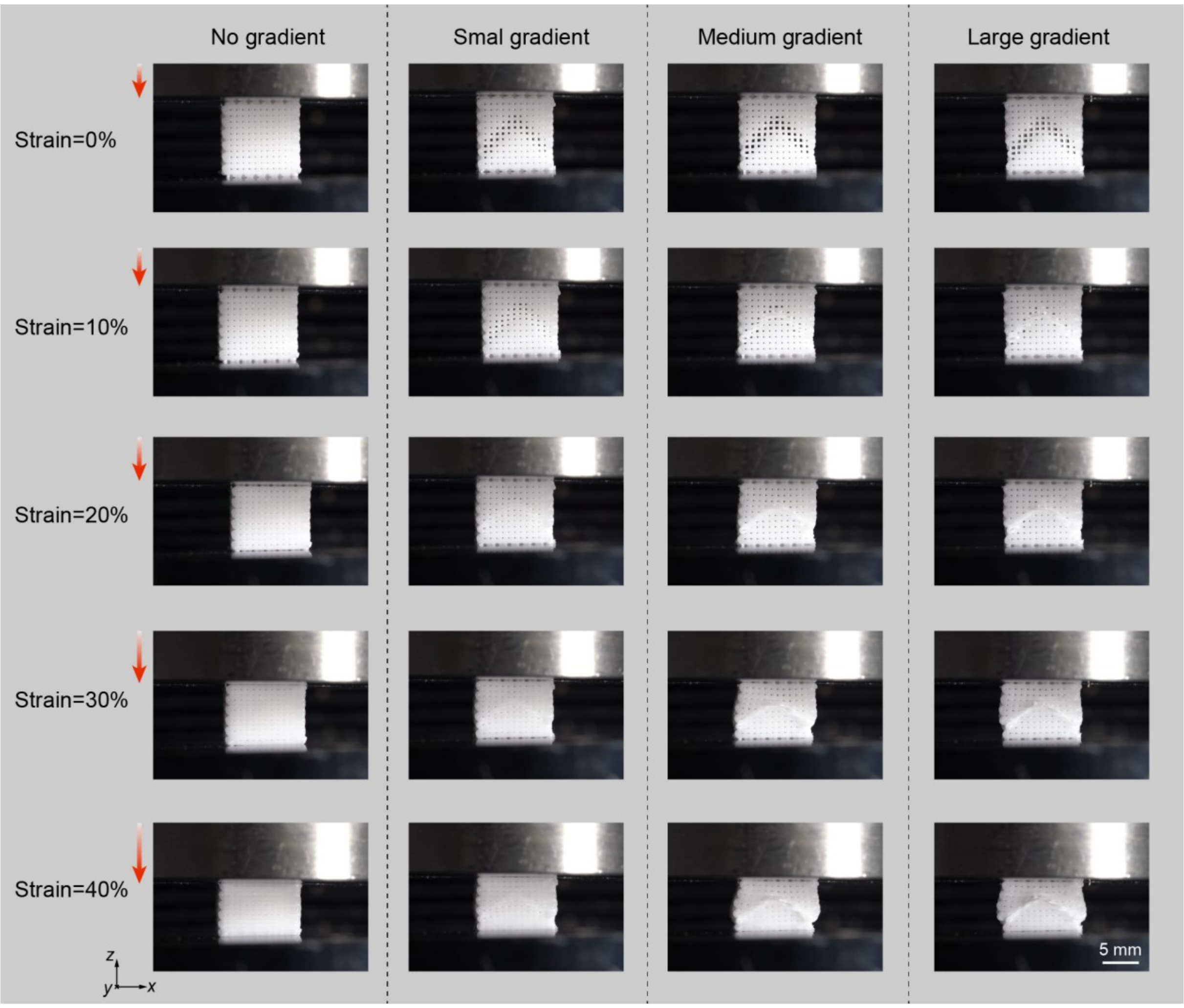

Supplementary Fig. 20 | Deformation of four samples with embedded V letter in uniaxial compression from 0% to 40% strain. The no gradient group does not have predictable regions of deformation during the compression process. Within ~20% strain, the V region of the three gradient groups is squeezed and the non-V region is not squeezed; after ~20% strain, the non-V region starts to be squeezed.

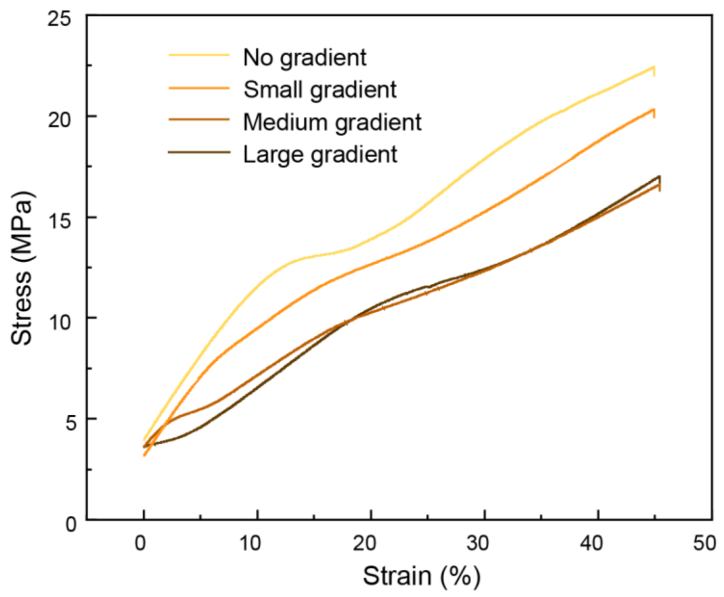

Supplementary Fig. 21 | Compression test of four V-shaped metastructures.

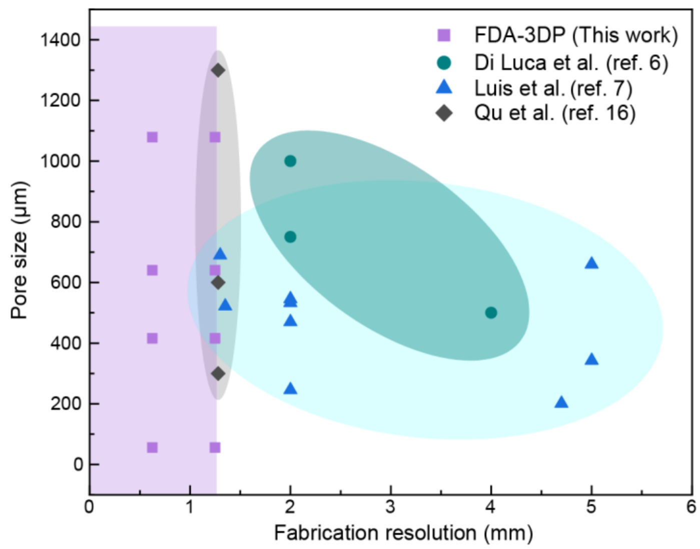

Supplementary Fig. 22 | Comparison of fabrication resolution and pore size between FDA-3DP strategy and reported methods for extrusion 3D printed radial gradient pore structures. Our proposed proposed in this work has high fabrication resolution (≤1.25 mm) and a wide range of pore diameter sizes (56-1079 μm). Di Luca et al.[6] and Luis et al.[7] divided the target model into different areas along the radial direction and filled them with variable diameters and spacing filaments. Qu et al.[16] proposed a fractal iterative method to design filling patterns and constructed radial gradient pores.

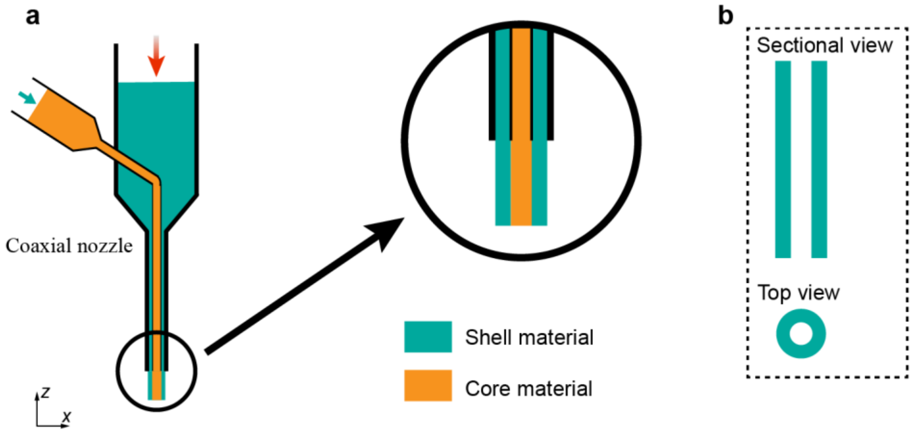

Supplementary Fig. 23 | Schematic diagram of coaxial nozzle. For preparing the vascular scaffold, GelMA and F127 are used as the shell and core materials, respectively (Fig. 6c); in the pressure sensor, PDMS and liquid metal (EGaIn) are used as the shell and core materials, respectively (Fig. 6d).

Supplementary Tables

Supplementary Table 1 | A comparison between the FDA-3DP strategy and the reported DIW 3D printing methods for creating gradient pore structures, focusing on gradient dimension, gradient resolution, and shape fidelity. A check mark (✓) denotes feasibility or compatibility, while a wrong mark (✗) denotes infeasibility or unattainability. A hyphen mark (-) indicates not mentioned in the literature.

Changing parameters	**Gradient dimension**[1]				**Gradient resolution**[2]	**Shape fidelity**[3]		Ref.
	1D			2D & 3D	Resolution level	Fidelity level	No collapse	
	Horizontal	Axial	Radial					
Diameter/spacing/angle within layers	✓	✗	✗	✗	Low	Low	✗	2,4,6,7
Diameter/spacing between layers	✗	✓	✗	✗	Medium	Medium	✓	3,5
Diameter/spacing in radial regions	✗	✗	✓	✗	Medium	Low	✗	6,7,16
Width of oblong filaments	✓	✓	✓	2D ✓<br>3D ✗	High	Low	✗	8-10
Diameter & selectively adding support layers	✓	✓	✓	✓	High	High	✓	**This work**

[1] Gradient dimension includes 1D, 2D, and 3D. 1D represents one direction of the *x-y-z* coordinate axis.
[2] Gradient resolution indicates the ability to create gradient pores.
[3] Shape fidelity is the difference between the designed model and the fabricated sample.

Supplementary Table 2 | Dimensional values for the horizontal gradient model and samples.

Three dimensional	CAD model	μ-CT-based 3D model (mm)				
		Sample 1	Sample 2	Sample 3	Average	Error
x-axis	10 mm	10.7	10.3	9.9	10.30	0.40
y-axis	10 mm	10.5	10.1	10.6	10.40	0.26
z-axis	10 mm	9.7	9.8	10.0	9.83	0.15

Supplementary Table 3 | Working parameters in Fig. 2.

Location	Ink material	Extrusion air pressure (kPa)	Nozzle inner diameter (μm)
In Fig. 2a-(ii)	H-PCL	500 kPa	400 μm
In Fig. 2a-(iii), 1st row	H-PCL	500 kPa	400 μm
In Fig. 2a-(iii), 2nd row	H-PCL	500 kPa	500 μm
In Fig. 2a-(iii), 3rd row	H-PCL	400 kPa	400 μm
In Fig. 2a-(iii), 4th row	L-PCL	400 kPa	400 μm
In Fig. 2b-d and f	H-PCL	500 kPa	400 μm

Supplementary Table 4 | Parameters of printed samples shown in this work. Coaxial printing (Fig. 4c-d) and 4D printing (Fig. 4e) were performed at room temperature (25 °C), and the remaining 3D printing (gray background area) was done at 110 °C and extrusion pressure of 500 kPa using H-PCL material. All samples were printed at 100% extrusion multiplier, i.e. full extrusion flow.

Location	Gradient	Model size (mm)	Fiber spacing (mm)	Printing velocity *V* (mm/s)	Nozzle height *H* (mm)	Pore size *D* (μm)
Fig. 1b	No	10×10×10	1.5	3	0.30	448
Fig. 1d & 3	Horizontal	10×10×10	1.5	2.1-13.1	0.33-0.18	0-1128
Fig. 2d	Linear	Length 20	-	Slow=3, Fast=8	0.4, 0.7	-
Fig. 2f	Linear	Length 40	-	0.5-13.1	0.33-1.65	-
Fig. 4	Axial	10×10×10	1.5	2.1-13.1	0.33-0.18	0-1176
	Point	10×10×10	1.5	2.1-13.1	0.33-0.18	0-1176
	Line					
	Surface					
	Body					
Fig. 5a	Through	20×10×10	0.83	Min=5, Max=13	0.25, 0.18	0-640
	Center					
	Cent. & Rot.					
Fig. 5b	No	10×10×10	0.82	5	0.25	210, 217
	Small	10×10×10	0.82	Min=5, Max=8	0.25, 0.22	215, 388
	Medium	10×10×10	0.82	Min=5, Max=11	0.25, 0.19	220, 486
	Large	10×10×10	0.82	Min=5, Max=14	0.25, 0.18	212, 514
Fig. 6a	Radial	φ10×15	1.5	2.1-13.1	0.33-0.18	0-1092
Fig. 6b	Radial	∠14.33°	Varying	2.1-13.1	0.33-0.18	377-943
Fig. 6c	Linear	Length 40	-	1.1-10	0.30-0.90	-
Fig. 6d	Linear	Length 25	-	0.75-2.9	0.5-1.30	-
Fig. 6e	Linear	Radius 15	-	1.1-10	0.30-0.90	-

Supplementary Table 5 | A comparison for creating a bone-mimicking scaffold between the FDA-3DP strategy and the reported methods. A check mark (✓) denotes feasibility or compatibility, while a wrong mark (✗) denotes infeasibility or unattainability. Repetition of symbols indicates degree.

Strategies	CAD model	Printed sample	Radial gradient	Features	Ref.
Triply-periodic minimal surfaces (TPMS)	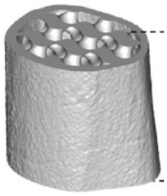	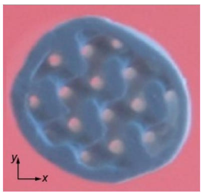	✗	Homogeneous pore and bionic cancellous-cortical bone	17
Conventional grid (0°/90°) via adjusting filament spacing between layers	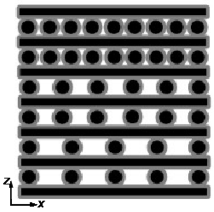	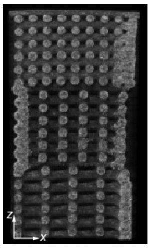	✗	Axial gradient	3
Conventional grid (0°/90°) via adjusting filament diameter in local regions	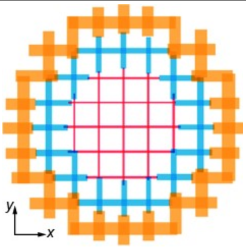	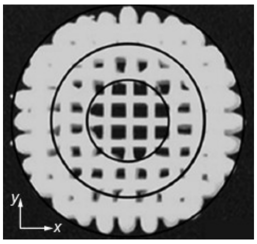	✓	Radial gradient, low gradient resolution, and height limitation	6,7
Fractal iterative pattern	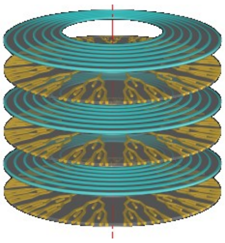	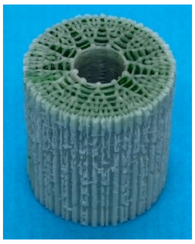	✓	Radial gradient, but low gradient resolution	16
FDA-3D printing strategy based on conventional grid (0°/90°)	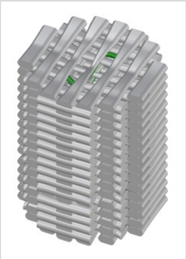	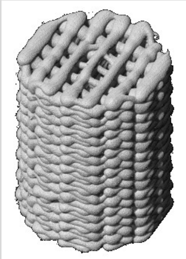	✓✓✓	Easy molding and highly tunable radial gradient	**This work**

Supplementary Table 6 | A comparison for creating a meniscus-mimicking scaffold between the FDA-3DP strategy and the reported methods. A check mark (✓) denotes feasibility or compatibility, while a wrong mark (✗) denotes infeasibility or unattainability. Repetition of symbols indicates degree.

Strategies	CAD model	Printed sample	Radial gradient	Features	Ref.
Conventional grid (0°/90°)	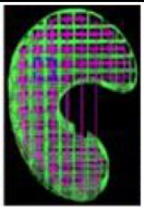	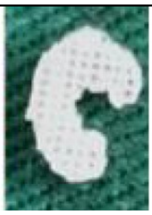	✗	No wedge cross section and no radial gradient	11
Conventional grid (0°/90°)	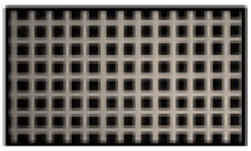	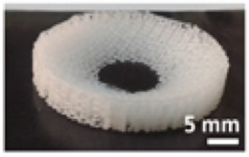	✗	No wedge cross section and no radial gradient	12
Radial and circular lines	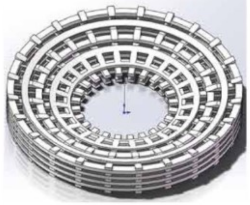	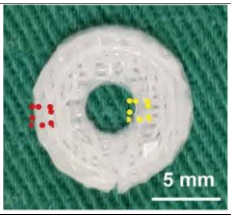	✓	Needs trimming and low tunable radial gradient	13
Grid (0°/90°) and radial lines	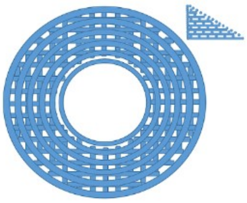	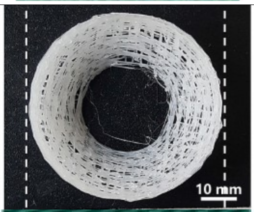	✓	Wedge tops difficult to fidelity and no radial gradient	14
Radial and circular lines	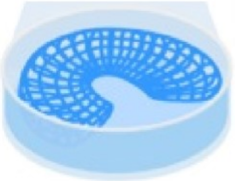	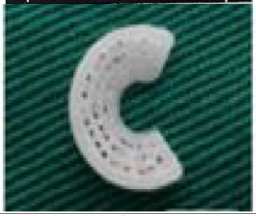	✓	Easy molding and low tunable radial gradient	15
Radial and circular lines	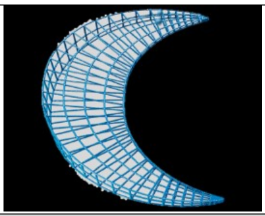	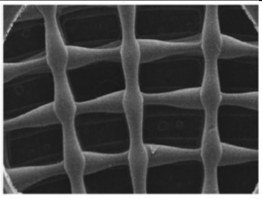	✓	Easy molding and low tunable radial gradient	1
Radial and circular lines via FDA-3D printing strategy	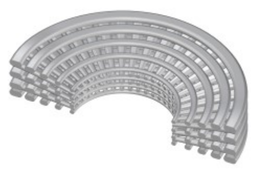	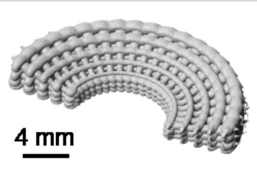	✓✓✓	Easy molding and highly tunable radial gradient	**This work**

References

1. Guo, W., et al. 3D-printed cell-free PCL–MECM scaffold with biomimetic micro-structure and micro-environment to enhance in situ meniscus regeneration. *Bioact. Mater.* **6**, 3620-3633 (2021).
2. Xie, C., et al. Structure-induced cell growth by 3D printing of heterogeneous scaffolds with ultrafine fibers. *Mater. Des.* **181**, 108092 (2019).
3. Bittner, S. M., et al. Fabrication and mechanical characterization of 3D printed vertical uniform and gradient scaffolds for bone and osteochondral tissue engineering. *Acta Biomater.* **90**, 37-48 (2019).
4. Shigueoka, M. O. & Volpato N. Expanding manufacturing strategies to advance in porous media planning with material extrusion additive manufacturing. *Addit. Manuf.* **38**, 101760 (2021).
5. Diloksumpan, P., et al. Orthotopic Bone Regeneration within 3D Printed Bioceramic Scaffolds with Region-Dependent Porosity Gradients in an Equine Model. *Adv. Healthc. Mater.* **9**, 1901807 (2020).
6. Di Luca, A., et al. Toward mimicking the bone structure: design of novel hierarchical scaffolds with a tailored radial porosity gradient. *Biofabrication* **8**, 045007 (2016).
7. Diaz-Gomez, L., Kontoyiannis P. D., Melchiorri A. J. & Mikos A. G. Three-dimensional printing of tissue engineering scaffolds with horizontal pore and composition gradients. *Tissue Eng. Part C-Methods* **25**, 411-420 (2019).
8. Yuk, H. & Zhao X. A New 3D Printing Strategy by Harnessing Deformation, Instability, and Fracture of Viscoelastic Inks. *Adv. Mater.* **30**, 1704028 (2018).
9. Moetazedian, A., Budisuharto A. S., Silberschmidt V. V. & Gleadall A. CONVEX (CONtinuously Varied EXtrusion): A new scale of design for additive manufacturing. *Addit. Manuf.* **37**, 101576 (2021).
10. Grigolato, L., Rosso S., Meneghello R., Concheri G. & Savio G. Design and manufacturing of graded density components by material extrusion technologies. *Addit. Manuf.* **57**, 102950 (2022).
11. Sun, Y., et al. 3D-bioprinting ready-to-implant anisotropic menisci recapitulate healthy meniscus phenotype and prevent secondary joint degeneration. *Theranostics* **11**, 5160-5173 (2021).
12. Bahcecioglu, G., Hasirci N., Bilgen B. & Hasirci V. A 3D printed PCL/hydrogel construct with zone-specific biochemical composition mimicking that of the meniscus. *Biofabrication* **11**, 025002 (2019).
13. Zhang, Z.-Z., et al. Orchestrated biomechanical, structural, and biochemical stimuli for engineering anisotropic meniscus. *Sci. Transl. Med.* **11**, eaao0750 (2019).
14. Bahcecioglu, G., Bilgen B., Hasirci N. & Hasirci V. Anatomical meniscus construct with zone specific biochemical composition and structural organization. *Biomaterials* **218**, 119361 (2019).
15. Li, Z., et al. Biomechanically, structurally and functionally meticulously tailored polycaprolactone/silk fibroin scaffold for meniscus regeneration. *Theranostics* **10**, 5090-5106 (2020).

16. Qu, H., et al. Fractal Design Boosts Extrusion-Based 3D Printing of Bone-Mimicking Radial-Gradient Scaffolds. *Research* **2021**, 9892689 (2021).
17. Charbonnier, B., et al. Custom-made macroporous bioceramic implants based on triply-periodic minimal surfaces for bone defects in load-bearing sites. *Acta Biomater.* **109**, 254-266 (2020).
