# Supplementary material for: Gradient matters via filament diameter-adjustable 3D printing
Source: Nat Commun. 2024 Apr 4;15:2930. doi: 10.1038/s41467-024-47360-y (PMC10994943; doi:10.1038/s41467-024-47360-y)
Supplement: Supplementary file 3 — Description of Additional Supplementary Files [file 41467_2024_47360_MOESM3_ESM.pdf]

Description of Additional Supplementary Files for

# Gradient matters via filament diameter-adjustable 3D printing

*Huawei Qu, Chongjian Gao, Kaizheng Liu, Hongya Fu, Zhiyuan Liu, Paul H. J. Kouwer, Zhenyu Han* & Changshun Ruan**

**Supplementary Video 1 |** Pore size maps of traditional- and FDA-3DP strategies. A horizontal gradient was chosen as an example to demonstrate the ability of the FDA-3DP strategy to create gradient pores. The video shows 2D images (*x-y* plane view) of printed samples and their pore size maps, and they appear gradually as the *z* height increases from bottom to top. The 2D images of printed samples were the μ-CT images, and their pore size maps were obtained by processing the μ-CT images using the software ImageJ. The samples obtained from the traditional- and FDA-3DP strategies have homogeneous and heterogeneous pore structures, respectively. The maximum pore size is 1128 μm.

**Supplementary Video 2 |** FDA-3D printing with constant printing height. A 20 mm-long printing trajectory is divided into two equal parts and set to two printing velocities ($V$ = 3 and 8 mm/s), but they have a fixed printing height ($H$ = 1.5 mm). During 3D printing, the first 10 mm-long trajectory shows an unacceptable rope coiling effect, and the last 10 mm-long trajectory presents an acceptable deposition state (see the constant $H$ group in Fig. 2d). The video is not accelerated or decelerated (1×).

**Supplementary Video 3 |** FDA-3D printing with variable printing height. A 20 mm-long printing trajectory is divided into two equal parts and set to two printing velocities ($V$ = 3 and 8 mm/s) and corresponding variable printing height ($H$ = 0.7 and 0.4 mm) based on the FDA-3DP strategy. During 3D printing, the whole 20 mm-long trajectory shows an acceptable and expected filament deposition state (see the variable $H$ group in Fig. 2d). The video is not accelerated or decelerated (1×).

**Supplementary Video 4 |** FDA-3D printing of horizontal gradient pore structure. The video shows the stacking process of 21 FDA layers and 8 supporting layers. The printing parameters in the video is from Fig. 2i. Here, the FDA layer filaments have variable $D$ (depending on pore

size gradient) and $H$; and the supporting layer filaments have a constant $D_{min}$. The video playback speed is fast forwarded four times (4×).

**Supplementary Video 5 |** Pore size maps of axial and radial gradient pore structures. The radial gradient includes point, line, surface, and body. The *x-y* intercept plane of the pore size map appears gradually as the *z* height increases from bottom to top. The result shows that the pore size of the axial sample gradually decreases from bottom to top; the pore size of the point-interfering sample is the largest in the center position, showing a regular radial gradient, and gradually decreases from the center to the upper and lower positions; the pore size of line-interfering sample has a consistent radial gradient from the bottom to the top; the pore size of the surface-interfering sample is the largest at the location of the cylindrical surface; the pore size of the body-interfering sample is large in the middle position and small in the upper and lower positions. 2D images (*x-y* plane) of pore size maps were obtained by processing the μ-CT images using the software ImageJ. The maximum pore size is 1176 μm.

**Supplementary Video 6 |** Pore size maps of gradient structures with embedded HIT letters. The positions of HIT letters (abbreviations of Harbin Institute of Technology) include penetration, centration, and centration & rotation. Notably, in the penetration group (lift), the full HIT letters can be viewed in all *x-y* planes of the pore size map from bottom to top. In the centration group (middle), the full HIT letters can only be observed in the middle position, not in the upper and lower positions. In the centration & rotation group (right), only the partial part of the HIT letters can be visualized in the middle position, not in the upper and lower positions. 2D images (*x-y* plane) of pore size maps were obtained by processing the μ-CT images using the software ImageJ. The maximum pore size is 640 μm.

**Supplementary Video 7 |** Compression test of metastructures. Three metastructures with embedded V letter have small, medium, and large gradients. A homogeneous pore sample is used as a control group. The video playback speed is fast forwarded four times (4×). Scale bar is 5 mm.

**Supplementary Video 8 |** Penetration of vascular scaffold. The upper scaffold has tunable width $l_w$ and uniform straightness, and the lower scaffold not only has a controllable width, but also can realize the controllable fluctuation. The perfusion medium is red dye water, and the

silver-white materials inside the scaffold are bubbles before the red ink enters. The video is played at normal speed (1×). Scale bar is 10 mm.

**Supplementary Video 9 |** FDA-4D printing. Octopus-mimicking alginate sample with eight tentacles was deposited on the substrate via the FDA-3DP strategy and then cross-linked using a 1.0 M calcium chloride solution ($Ca^{2+}$) for 30 seconds. The cross-linked alginate sample was left at room temperature (25 °C). As water evaporated, eight tentacles gradually bent downward, eventually supporting the entire sample after about 1 hour. The video playback speed is fast forwarded a hundred times (100×). Scale bar is 5 mm.

**Supplementary Code 1 |** G-code file was used to FDA 3D print the horizontal gradient porous sample.
