# Supplementary material for: Gradient matters via filament diameter-adjustable 3D printing
Source: Nat Commun. 2024 Apr 4;15:2930. doi: 10.1038/s41467-024-47360-y (PMC10994943; doi:10.1038/s41467-024-47360-y)
Supplement: Supplementary file 13 — Supplementary Code 1 [file 41467_2024_47360_MOESM13_ESM.zip › G-codes/ReadMe.docx]

This zip includes the word file (ReadMe.docx) and the G-code file for filament diameter-adjustable (FDA) 3D printing (Horizontal_Gradient_Porous_Structure.gcode), designed by Huawei Qu *et al.* from Shenzhen Institute of Advanced Technology, Chinese Academy of Sciences. As a proof of concept, in this zip, we provide the customized fabrication file (G-codes) for the horizontal gradient porous scaffold. The design and printing process for the horizontal gradient porous sample is shown in Fig. 3 and Supplementary Video 4 (Our Manuscript entitled “*Gradient matters via filament diameter-adjustable 3D printing*”). We employ a workstation desktop computer (Precision T5820, Dell Inc., USA) with a Windows 10 system.

The steps to note are as follows:

1. Prepare the viscoelastic ink H-PCL, as described in the Methods section.
2. Initialize the commercially available extrusion-based 3D printer (Regenovo Bio-Architect WS, Hangzhou Regenovo Biotechnology Co., Ltd., China).
3. Use the control software corresponding to the Regenovo 3D printer (3D Bio-Architect, Hangzhou Regenovo Biotechnology Co., Ltd., China) to execute our customized fabrication file (G-codes) (i.e., Horizontal_Gradient_Porous_Structure.gcode) for the FDA-3D printing.
